# Properties of the cuticular proteins of *Anopheles gambiae* as revealed by serial extraction of adults

**DOI:** 10.1371/journal.pone.0175423

**Published:** 2017-04-18

**Authors:** Yihong Zhou, Majors J. Badgett, Lynne Billard, John Hunter Bowen, Ron Orlando, Judith H. Willis

**Affiliations:** 1 Department of Cellular Biology, University of Georgia, Athens, Georgia, United States of America; 2 Complex Carbohydrate Research Center, University of Georgia, Athens, Georgia, United States of America; 3 Department of Statistics, University of Georgia, Athens, Georgia, United States of America; Texas A&M University College Station, UNITED STATES

## Abstract

How cuticular proteins (CPs) interact with chitin and with each other in the cuticle remains unresolved. We employed LC-MS/MS to identify CPs from 5–6 day-old adults of *Anopheles gambiae* released after serial extraction with PBS, EDTA, 2-8M urea, and SDS as well as those that remained unextracted. Results were compared to published data on time of transcript abundance, localization of proteins within structures and within the cuticle, as well as properties of individual proteins, length, pI, percent histidine, tyrosine, glutamine, and number of AAP[A/V/L] repeats. Thirteen proteins were solubilized completely, all were CPRs, most belonging to the RR-1 group. Eleven CPs were identified in both soluble fractions and the final pellet, including 5 from other CP families. Forty-three were only detected from the final pellet. These included CPRs and members of the CPAP1, CPF, CPFL, CPLCA, CPLCG, CPLCP, and TWDL families, as well as several low complexity CPs, not assigned to families and named CPLX. For a given protein, many histidines or tyrosines or glutamines appear to be potential participants in cross-linking since we could not identify any peptide bearing these residues that was consistently absent. We failed to recover peptides from the amino-terminus of any CP. Whether this implicates that location in sclerotization or some modification that prevents detection is not known. Soluble CPRs had lower isoelectric points than those that remained in the final pellet; most members of other CP families had isoelectric points of 8 or higher. Obviously, techniques beyond analysis of differential solubility will be needed to learn how CPs interact with each other and with chitin.

## Introduction

Thousands of putative cuticular protein (CP) sequences are now available with well over 100 from many individual insect species. While most proteins designated as “cuticular” are based on sequence similarity to previously identified CPs, an increasing number have been verified as authentic via mass spectrophotometry analyses of cuticle or cuticle-rich structures. We recently confirmed the authenticity of 82% (230/282) of the different CPs from *Anopheles gambiae* by combining results with tandem mass spectrometry from our laboratory with that from three other studies [1]. Now we need to learn more about the properties of these proteins to facilitate unravelling how they are deployed in forming the diverse cuticle types found even in a single species.

A good starting point is chitin-binding and two main methods have been employed to this end. In the first, individual CPs have been expressed *in vitro*, frequently conjugated to another protein such as glutathione S-transferase, and tested for binding to purified chitin [2–6]. That method established that the members of the largest family of CPs in every species examined to date, the CPR family, bind chitin via an extended version of the R&R Consensus that gives the family its name. The Consensus was first recognized by Rebers and Riddiford [7] in 6 CPs and subsequently has been extended towards the N-terminus and shortened towards the C-terminus. It now has about 53 amino acid residues and is recognized as pf00379 or chitin_bind 4.

The second method used to assess chitin-binding is to extract proteins from cuticle preparations, remove the solubilizing agent, subject the extract to chitin, and then use some form of mass spectrometry to identify the proteins that bind [5, 8].

Both of these methods have a critical problem: they measure chitin-binding under conditions that in no way resemble the intra-cuticular environment. Normally chitin and proteins interact immediately after secretion in the assembly zone or inner regions of the procuticle. The relative concentrations of proteins and chitin, as well as the pH and salt concentration of this specialized environment, are not mimicked by conditions of *in vitro* chitin-binding. Furthermore, the chitin used for binding is certainly not in the same configuration as that found for chitin during cuticle assembly. In addition, the finding of chitin deacetylase in cuticle preparations [9] indicates that we need to learn about proteins that bind deacetylated chitin as well as chitin. Salt concentration appears to be critical for chitin-binding as Rebers and Willis [2] had to use 0.5M NaCl to avoid background with glutathione S-transferase (GST) to which they had fused the Consensus, while Togawa et al. [3] used 20 mM sodium phosphate and reported 30% background binding of GST alone.

We sought another way to assess the behavior of proteins within the cuticle, namely subjecting pulverized cuticle or even pulverized whole animals to a regimen of increasing strength of compounds known to affect chitin-binding. This method was used decades ago, but at a time when amino acid sequences for individual proteins were not known and protein identification had to be based on the position of an electrophoretic band or the amino acid composition of fractions that were eluted under different conditions. Hackman [10] is the first paper to report the use of gel electrophoresis to identify proteins rendered soluble by different solvents and has references to earlier studies. Now, at last, it is possible to employ this procedure of serial extraction to learn precisely which proteins are solubilized with different solvents. While this method lacks the specificity of looking for chitin-binding, it has the advantage of assessing **all** cuticular proteins including those that lack known chitin-binding domains or cross-linking motifs. It enables one to learn which CPs have been rendered insoluble in the process of cuticle maturation. Furthermore, analysis of the peptides released by trypsin from the residue that remains after the solvents have been deployed should enable us to learn if any regions of a protein have been modified by cross-linking, rendering peptides containing them undetectable. We were particularly interested in two chitin-binding domains recognized at the pfam site (http://pfam.xfam.org/): the extended R&R Consensus (pf00379 or chitin_bind_4) and the chitin-binding domains of the CPAPs (pf01607 or CBM_14 or chitin-binding peritrophin A domain). We may obtain information about regions with histidine and lysine residues that might be involved in quinone cross-links, tyrosines that could be cross-linked by peroxidase, bonds formed between glutamines and lysines by the action of transglutaminase, or the short motifs containing GYR or YLP that Cornman [11] suggested might also be involved in protein/protein association.

We chose to work with *An*. *gambiae* because of the information already available about its 282 CPs. Data in our original LC-MS/MS were obtained from what we hoped were relatively pure cuticle preparations: the head capsule shed as 4^th^ instar larvae molted to pupae, and the pupal cuticle left behind after ecdysis [12]. Both of these preparations, however, have been subjected to molting fluid, so it is likely that some CPs had been removed by proteolytic action and chitinases in the molting fluid may have altered the chitin. For this study, we chose to begin with intact adults. Our previous work with individual adult structures, legs, wings, corneal lens, and antennae, showed that the most abundant proteins extracted from these structures with 1% SDS or that remained in the final pellet had been annotated previously as cuticular proteins [1].

We now report on the serial extraction of proteins from 5–6 d old adult mosquitoes. The treatments are described in detail in **Materials and methods**. Basically, after grinding in liquid nitrogen, we extracted proteins, first with potassium tetraborate to remove non-cuticular proteins, then in PBS, followed by EDTA, then with increasing concentrations of urea, and finally with SDS. These samples, plus the final pellet remaining after completion of all extractions, were treated with trypsin and analyzed with LC-MS/MS to identify the peptides released. While we acknowledge that the initial preparatory steps are giving us material somewhat removed from the natural state, it is certainly closer to the native condition than either recombinant proteins or those acquired by extraction and renaturation.

We have annotated 164 genes that code for proteins with the chitin-binding extended R&R Consensus (pf00379). This region has been subdivided into three groups RR-1, RR-2 and RR-3 [13, 14] and described in detail in [15]. We were interested in learning whether these different groups differed in their extractability. There are several other families of CPs in *An*. *gambiae* that have been shown to bind chitin, the CPAP1 and CPAP3 families that have one and three copies, respectively of a well characterized chitin-binding domain, pf01607 [16–18]. In *Bombyx mori*, one member of the TWDL family (CPT1) was shown to bind to chitin [5]. Quite recently, Dong et al. [8] used the extract, renature, chitin-bind protocol on proteins extracted from the cleaned cuticle of day 5 final instar larvae of *Bombyx* and identified CPs bound to chitin. Considering only the CP families present in *An*. *gambiae*, they found binding of two CPTs (TWDLs), 36 CPRs plus 6 that had indistinguishable partners, 3 CPAP1s and 5 CPAP3s, and for the first time found binding for 2 CPFLs and the only *Bombyx* CPCFC.

The total number of CP genes we have identified in *An*. *gambiae* is now 295. Many genes, especially those in the RR-2 group, are found in sequence clusters, associations of genes with very similar, and sometimes identical, protein sequences [19]. There are also identical sequences or proteins with many shared peptides found among CPLCGs, CPLCPs and CPLCWs. Hence, there are only 282 unique proteins in the CP repertoire of *An*. *gambiae*. Our serial extraction data should give us information about the relative ease with which each of these proteins, if present in the cuticle of adults, can be extracted and which peptides fail to appear in the data for each extraction method. Furthermore, we will be able to assess the solubility of CPs with chitin-binding domains relative to those that appear to lack such domains.

Also relevant to the current analysis are results from electron microscopy (EM) immunolocalization of CPs within the cuticle itself [15, 20–22], for these will allow us to learn if location in the cuticle is related to solubility. A LC-MS/MS analysis on five adult structures, consisting of the second antennal segment with JO, the rest of the antenna, corneal lens, legs and wings, revealed which proteins are most abundant in the different structures and which were found in all structures [1]. Those data too should help us interpret the current findings.

Finally, we have RT-qPCR data for most CPRs [23], for CPFs and CPFLs [4] and several of the other families [20, 21, 24] as well as RNA-Seq data on 5 d old adult females [25]. These data should help us to learn if there is an association between the time when a CP transcript is present and the ability of the corresponding protein to be extracted. Finally, we can learn if any CPs with abundant transcripts in pharate or young adults fail to appear in the proteins we have recovered for that might indicate extensive modification in the course of cuticle maturation.

## Materials and methods

### Mosquito rearing

*An*. *gambiae* (G3 strain) were obtained from the insectary at the University of Georgia Entomology Department. Newly hatched first instar larvae were reared in our lab at 27°C in a 12/12 L/D photoperiod. Larvae were fed with ground Koi food Staple Diet (Foster and Smith Aquatics, Rhinelander, WI USA). Adults were returned to the insectary with an 18:6 photoperiod, higher humidity than in our incubator and supplied with water and an 8% fructose solution. Adults of both sexes were frozen at -80°C 5–6 days after eclosion. A recent paper [26] showed that storage of locust tibia at -20°C had no impact on their biomechanical properties, so our serial extraction process began with unaltered cuticles.

### Serial extraction of proteins

Three biological replicates of serial protein extraction (batches 1–3) were performed. Frozen *An*. *gambiae* adults of both sexes (n = 1100–1500) were pulverized in liquid nitrogen with mortar and pestle. The tissue powder was transferred to a 40 mL autoclaved Beckman centrifuge tube, We first washed the tissue powder three times with a 1% (w/v) potassium tetraborate solution containing 1% ascorbic acid (pH 7.7) and 0.5% Triton X-100. Potassium tetraborate solution can dissolve or soften most of the non-cuticular material in the cuticle, protect *o*-diphenols against oxidation, and thus has been used to prepare samples enriched in cuticle from various insects (See **H1**).

Serial extraction began with PBS (phosphate buffered saline, pH 7.4) followed by 0.1 M EDTA, 2 M, 4 M, and 8 M urea solutions. The antibiotic gentamycin (25 μg/mL) was added to the potassium tetraborate and PBS solutions to thwart bacterial growth. The EDTA and urea solutions were made in PBS. Buffering by PBS was insufficient to control the pH that was 0.4 units higher in 8M urea than in 2M (Table 1). Serial extractions were carried out twice for PBS, EDTA and the urea solutions. Tubes were shaken vigorously with an Orbit Shaker (Lab-Line) in a 6°C cold room and then centrifuged at room temperature in a Beckman J2-21 centrifuge with a JA-20 rotor (Beckman Instruments Inc., Palo Alto, California) at 18K RPM (39,000 xG) for 10 min.

**Table 1:** Time and volume of different solutions used for serial extraction of proteins.

Solution	Time of 1^st^ extraction/Vol (hrs/ml)			Time of 2^nd^extraction/Vol (hrs/ml)			Time of 3^rd^ extraction/Vol (hrs/ml)			^a^Quick washes (min/mL
	Batch 1	Batch 2	Batch 3	Batch 1	Batch 2	Batch 3	Batch 1	Batch 2	Batch 3	
Potassium tetraborate	3 / 20	6 / 20	6.5 / 20	16 / 20	16 / 20	16 / 20	4 / 15	7 / 15	6.5 /15	5 / 15
PBS pH 7.4	17.5 / 15	16 / 15	15.5 / 15	4 / 10	7 / 10	6 / 10	-	-	-	5 / 10
EDTA	16.5 / 8	16 / 8	16 / 8	6 / 8	7.5 / 8	7 / 8	-	-	-	5 / 8
2M urea pH7.5	16.5 / 8	16 / 8	15.5 / 8	7.5 / 8	7 / 8	9 / 8	-	-	-	5 / 8
4M urea pH7.6	15.5 / 8	17 / 8	14.5 / 8	6.5 / 8	7 / 8	7.5 / 8	-	-	-	5 / 8
8M urea pH7.9	16.5 / 8	15.5 / 8	17 / 8	6 / 8	8 / 8	9 / 8	-	-	-	5 / 8
1% SDS	0.3 / 5	0.3 / 5	0.3 / 5	0.3 / 5	0.3 / 5	0.3 / 5	0.3 / 5	0.3 / 5	0.3 / 5	5 / 3

^a^Final pellets after SDS extraction were washed 3 times with ddH_2_O. Pellets after extraction with other solutions were washed twice with the same solution that had been used for protein extraction.

The supernatant was transferred to a new tube, a fresh solution added and the pellet was dispersed thoroughly using an Ultra-turrax (Janke & Kunkel IKA-WERK) fitted with sharp blades. The duration and volume for each extract are shown in Table 1. Before each change to a new solution, the pellet was quickly washed twice with the same solution, following the same dispersing and centrifuging protocol. The pellet after 8M urea treatment was incubated with 5 mL 1% SDS containing 50 mM dithiothreitol (DTT) in a boiling water bath for 20 min (repeated 3 times). The final pellet was washed 3 times in 3 mL ddH_2_O and stored at -20°C, along with all the previous extracts and washes.

### Preparation of samples for LC-MS/MS

The protein concentration of the extract was estimated with a NanoDrop N-1000 (Thermo Scientific, USA) using the protein A280 method. Two μL of each extract were used for three replicate measurements. The quantity and protein profile of each extract were also examined using NuPAGE 4–12% Bis-Tris Gels (Cat. No. NP0323BOX, Invitrogen). Gel images were taken with FluorChem 8900 imaging system (Alpha Innotech, San Leandro, CA). With all solutions, the first extract had the most material and was the only one used to prepare protein samples for LC-MS/MS analysis. The original extracts were concentrated (5–13 times, depending on the original concentration and volume), using Vivaspin columns (Sartorius) or a SpeedVac (Savant Instruments Inc). Concentrated extracts were resolved with the same NuPAGE gels with three identical aliquots (20 μL per lane) of the same sample placed in adjacent lanes. Gels were stained either with Generon Quick Coomassie Stain (Generon Maidenhead, UK) or Bio-Safe^™^ Coomassie G-250 Stain (Bio-Rad). The gels were run at a constant voltage of 100 volts for about 15 min when the entire gel region containing the proteins was used for LC-MS/MS analysis and for 45 min after which the gel area containing the proteins was horizontally divided into two or four equal parts prior to LC-MS/MS analysis (S1 Photos Panel D). Each gel sample was cut into small pieces (~ 1 mm^2^), then destained by sequential rinses with water (once), 50% acetonitrile (ACN) in water (twice), 25 mM NH_4_HCO_3_ (once) and twice with ACN. Alkylation and reduction were carried out by incubating the gel pieces in 10 mM DTT (dithiothreitol)//25 mM NH_4_HCO_3_ at 65°C for 1 h, followed by 55 mM IDA (iodoacetamide)/25 mM NH_4_HCO_3_ in the dark for 1 h. The pieces were washed with 25 mM NH_4_HCO_3_, dehydrated with ACN, and then incubated with sequencing grade trypsin (Promega) (50:1 w/w protein/trypsin) overnight at 37°C. The supernatant was transferred to a new tube; 50% ACN /0.1% formic acid was added to the gel pieces to further extract the digested peptides. Pooled supernatants were dried in a Speed Vac and re-dissolved in 20 μL of 5% ACN/0.1% formic acid solution before LC–MS/MS analysis.

The pellets left after SDS extraction and water washes were washed 5 times with 25 mM ammonium bicarbonate, reduced with 10 mM DTT, alkylated with 55 mM IDA, digested with trypsin (50:1 w/w protein/trypsin) overnight and then filtered through a 30 kDA spin filter (EMB Millipore). Supernatants were dried and re-suspended in 5% ACN/0.1% formic acid.

ACN can precipitate large proteins in the extracts and thus increase the concentration of the small proteins in the sample [27]. Most *An*. *gambiae* cuticular proteins have low molecular masses, so in addition to using the concentrated extracts directly for MS analysis, we also treated these concentrated extracts with ACN to increase the probability of detecting rare cuticular proteins in our samples. For each of the solutions, 500 μL concentrated extract was treated with ACN, following the protocol of Zhang et al. [27]. The final supernatants were transferred to new tubes and dried in a SpeedVac. The dried samples were redissolved in 100–200 μL ddH_2_O. Three identical aliquots (20 μL per lane) of the ACN-treated samples were run on the NuPAGE gel for 20 min (S1 Photos Panel E), and the entire gel region containing proteins was used for the LC-MS/MS analysis, applying the same protocol as for the other gel samples. The various types of samples prepared from each extract and used for LC-MS/MS analysis are shown in S1 Table.

### LC-MS/MS analysis

All samples were suspended in 15 μL of a solution of 0.1% formic acid/10mM ammonium formate and 1 μL of 80% ACN/0.1% formic acid/10 mM ammonium formate. Samples from batch 1 and 2 were run on the Orbitrap Elite Mass Spectrometer (Thermo-Fisher) and a Dionex Ultimate 3000 Series LC System (Thermo-Fisher) with a nanospray ionization source. Two μL of each sample were injected into the LC. Peptides were separated using a 75 μm x 150 mm Acclaim PepMap RSLC column packed with 2 μm diameter superficially porous particles (Thermo-Fisher). The gradient used for each sample was 4–65% of 80% ACN/0.1% formic acid over 94 min at a 300nl/min flow rate. The settings for the mass spectrometer included taking the 5 most intense ions from each full mass spectrum for fragmentation using CID (collision-induced dissociation), and the resulting MS/MS spectra were recorded.

All samples from batch 3 were run on an Orbitrap Fusion Tribrid Mass Spectrometer (Thermo Scientific). After resuspension the samples were racked into an Ultimate 3000 LC System (Thermo Scientific). LC-MS/MS analysis was performed utilizing a nanospray ionization source. Six μL of each sample were injected and separated using a 75-μm x 150-mm Acclaim PepMap RSLC column packed with 2-μm diameter superficially porous particles (Thermo Scientific). The gradient used was 0–55% of 80% ACN/0.1% formic acid over 90 min at a flow rate of approximately 300 nL/min. The top 20 most intense ions were fragmented with CID and the resulting MS/MS spectra were recorded. Dynamic exclusion was utilized to exclude precursor ions from the selection process.

### Database searching

Raw tandem mass spectra were converted using the Trans-Proteomic Pipeline (Seattle Proteome Center). MS/MS spectra of each sample were searched using Mascot (Matrix Scientific, Boston, MA, USA) against target and decoy protein databases. We searched against two databases, *An*. *gambiae* peptides (AgamP4.2) from VectorBase [https://www.vectorbase.org/downloadinfo/anopheles-gambiae-pestpeptidesagamp42fagz] and a locally constructed database of 304 putative and previously verified structural cuticular proteins (available at: ftp://massive.ucsd.edu/MSV000080580/sequence/Agam_CPs_071714.fasta). The use of a small database focused on expected proteins was recommended in [28] and was used in a previous publication from our laboratory [1]. Decoy databases were created by reversing the protein sequences of corresponding target databases.

The following parameters were utilized in Mascot searching: a fragment tolerance of 0.6 Da; a peptide tolerance of 100 ppm; monoisotopic mass search; a maximum of two missed cleavages by trypsin; a fixed modification of carbamidomethylation of cysteine; and variable modifications of oxidation of methionine and deamidation of asparagine or glutamine. Resulting Mascot files were analyzed using ProteoIQ (Premiere Biosoft; http://www.premierbiosoft.com/protein_quantification_software/index.html), where a 5% false discovery rate (FDR) was employed for confirmation of protein identifications.

All data have been submitted to MassIVE and ProteomeXchange with the following accession numbers: MSV000080580 and PXD005983.

### Statistical analyses

**Fig 1:**
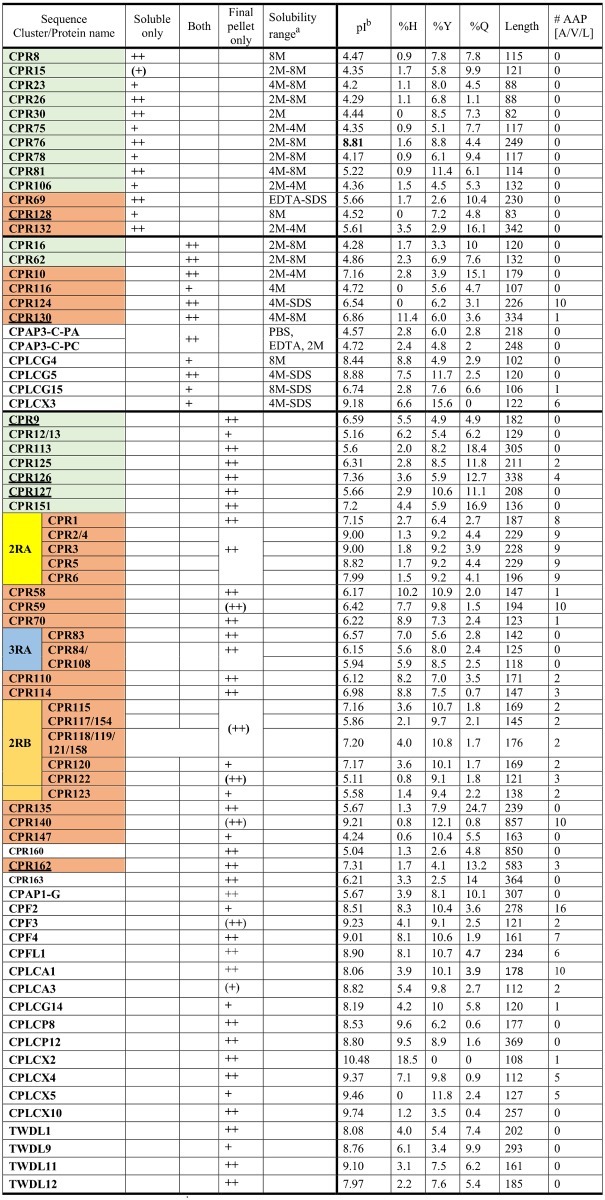
Solubility classes and properties of CPs identified in extracts and final pellet. Data in the first 5 columns summarize data from this analysis. Parentheses around a + indicate that assignment to solubility group had minor exceptions. Proteins identified with three or more peptides are indicated with ++. Data entry required that at least one peptide for a protein was found in two of the three biological replicates. Details are in S2 Table. Names of CPR proteins in the RR-1 group have a green background, RR-2 have a brown background. Names underlined scored below recommended threshold in CutProtFam-Pred. Two CPR sequences in small type and with no background color, did not score as either group but had a region recognized as pfam00379. Protein names separated by a / had identical sequences. ^a^Data from S2 Table. ^b^Isoelectric points obtained from (http://web.expasy.org/compute_pi/).

Analyses were carried out on the data in Fig 1 and S3 Table using analyses of variance F tests carried out on SAS PROC GLM. Chi-square tests were done using SAS.

## Results and discussion

### Overview

The rationale for this project was to learn if different CPs either as individuals or as members of families could be classified based on their extractability from frozen and pulverized 5–6 d old adult *An*. *gambiae*. To assure reproducibility, we repeated the entire extraction protocol three times. The pulverized animals had a preliminary treatment with potassium tetraborate that had been shown to remove non-CPs (see **H1**), and were then subjected to six different solvents, each two times followed by rinses. The first extracts from each of these treatments were run into gels and the peptides recovered from one or four sections of these gels after treatment with trypsin were analyzed by LC-MS/MS. (See S1 Photos Panels D and E for illustration.) The final pellet was also trypsinized and the resulting peptides analyzed.

We also treated some of the fractions with ACN before running in a gel to enrich for low molecular weight proteins (S1 Table). The efficacy of the ACN treatment is shown in S1 Photos Panel C. Although ACN was effective in eliminating the proteins with higher molecular masses, only one unique protein was recovered relative to the untreated fraction (S9 Table), and this protein had also been found in the final pellet. Out of 16 peptides recovered in the ACN extracts but not in the corresponding untreated sample, only five peptides had not been found in other samples (S2 and S9 Tables). Hence, ACN treatment had a very minor influence on peptide detection.

All recovered peptides organized by solubility class and then protein are in S2 Table where we have highlighted the 68 proteins with peptides recovered in a particular fraction in at least two of the three replicates; these data are summarized in Fig 1 and discussed below. S2 Table also gives the AGAP numbers assigned by VectorBase for each protein. In total, we recovered 418 peptides from 83 individual CPs. There were 51 peptides from 20 proteins and 11 sets that failed to reach our criterion either because they belonged to two or more CPs or because there were insufficient replicates. Although we searched the MS/MS results using Mascot with two different databases, the vast majority of the CP peptides recovered were found in both searches, and the differences are shown in S2 Table. The peptides mapped to their respective proteins are shown in S1, S2, S3 and S4 Files. Data for non CPs will be discussed in a final section.

**Fig 2:**
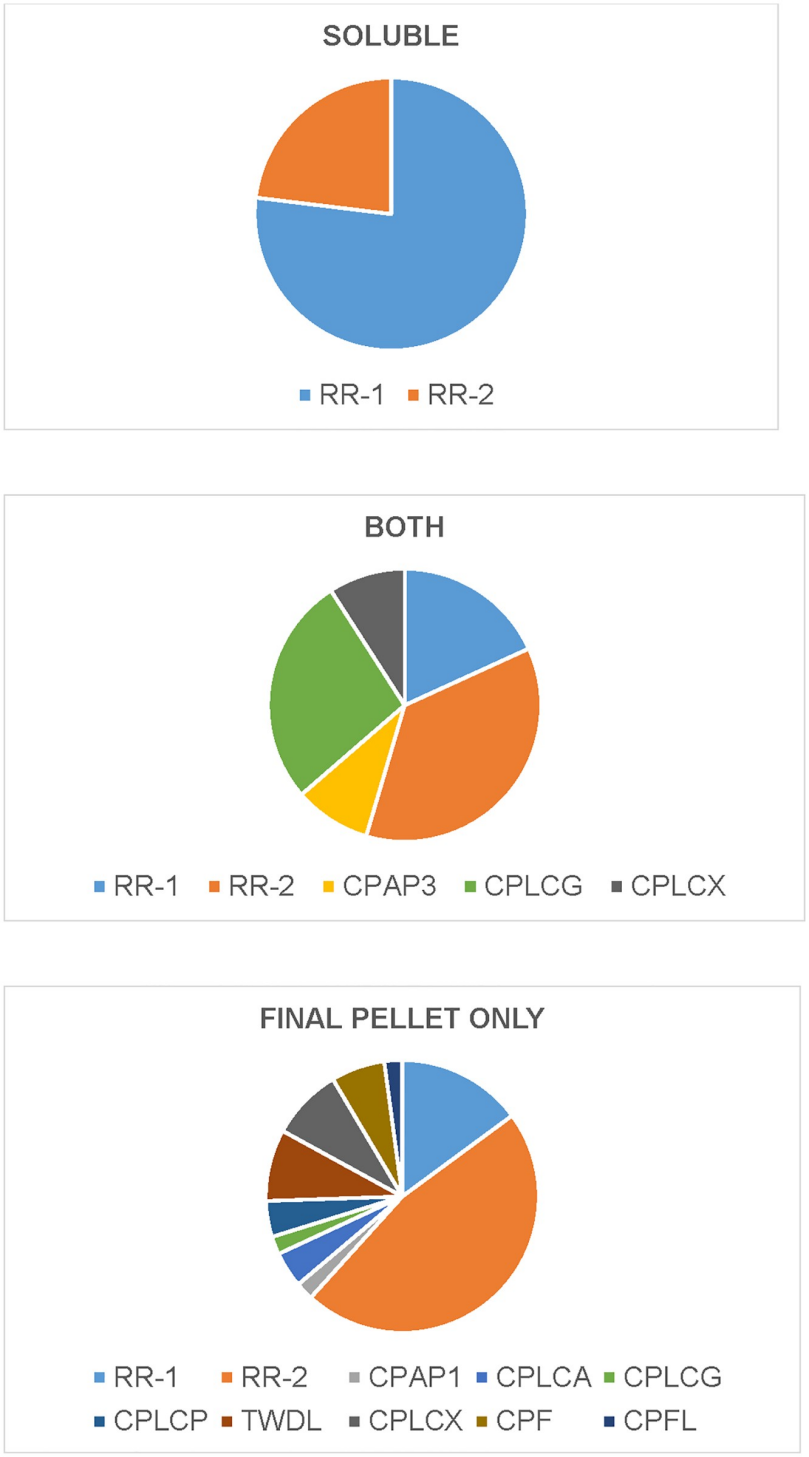
Graphical depiction of the distribution of different CP families in the soluble, soluble + final pellet, and final pellet fractions.

Fig 1 organizes the solubility categories. Seven proteins had an occasional peptide in a solubility class different from the majority. They are shown in parentheses in Fig 1 but are included in the counts. There was one set of 13 proteins that were recovered exclusively or predominantly in soluble fractions. A second set of 11 was recovered from both extracts and the final pellet. Peptides for most proteins were only recovered in the final pellet that had 42 proteins with unique peptides and 7 more, members of sequence clusters, with only shared peptides. Although many protein bands were present in the EDTA extracts (S1 Photos Panel B), there were only 3 peptides from 2 CPs recovered from that preparation (Fig 1, S2 Table). A summary of the distribution of protein families in the different fractions is in Fig 2.

### Tests of hypotheses relevant to understanding solubility properties of CPs

Our analysis was aided by data of several types we have published since we began to study the CPs of *An*. *gambiae*. We were able to pose several specific hypotheses about possible associations with solubility class and have organized the paper to address these hypotheses. This method of organization fits with the recent suggestion that one should consider “a forest of hypotheses” [29].

#### H1—Some CPs were extracted and discarded during the preliminary treatment with potassium tetraborate

We used preliminary extraction with potassium tetraborate, a method first introduced by Andersen [30] that should preferentially eliminate non-CPs. Andersen justified this treatment by stating: “…it also removes all water-soluble low molecular weight compounds, whereas both the sclerotized and non-sclerotized proteins in the cuticle are unaffected.” The method has subsequently been widely used, generally as an aid in manual cleaning of cuticle (early references are [31–33], but it has also been used to obtain material enriched in cuticular components from whole animals, including blowflies [34] and mosquitoes [35].

Electrophoretic gels revealed, as expected, that many proteins were present in the tetraborate extracts and these were completely extracted by the third treatment (S1 Photos Panel A). We wondered if some were CPs that would have been lost from subsequent analysis. Thus we compared data we obtained from our current results with those from a LC-MS/MS analysis of SDS extracts and final pellets from five different structures from adult *An*. *gambiae* [1]. These samples had been extracted directly in SDS without a prior rinsing step and thus the extract plus final pellet should contain all CPs in those structures. The only proteins that we had not found in our current analysis had been detected by no more than two peptides and from only one or two structures. Thus they would be minor proteins in extracts of whole animals.

An independent way of assessing possible protein loss is to look at the expression profiles of CPs. RT-qPCR has been done for the first 156 CPRs [23], for all CPFs and CPFLs [4], and for many of three families of the cuticular proteins with low complexity, CPLCs [24]. We discuss these data in detail in a subsequent section in reference to proteins we had recovered. We did not find any genes with high transcript levels in pharate adults or young adults for which we had not recovered proteins. Thus it is unlikely that our preliminary rinsing step resulted in our discarding a significant number of CPs.

#### H2—There is an association between CP family assignment and protein solubility

The most abundant CP family in every insect species examined to date is the CPR family. We have annotated 164 members in *An*. *gambiae*, but because of mature protein sequence identity, there are only 155 distinct protein sequences. We recovered peptides from 43 distinct CPR sequences and an additional 7 that belonged to sequence clusters (Fig 1, S2 Table). There was a clear association between CP family and solubility (p = .0001). Ten out of 13 proteins in the soluble fractions were RR-1s, far more than the 3 expected by chance alone and there were fewer than expected RR-1s in the final pellet.(S8 Table). Almost half of these proteins started to be extracted in 2M urea, but solubility continued at higher concentrations, suggesting some sort of differential binding to chitin or more likely the complex nature of urea solubilization [36–38]. Of the soluble proteins, only CPR8 (RR-1), CPR128 (RR-2), and CPLCG4 required 8M urea for solubilization (Fig 1). Boiling SDS did not increase the number of proteins recovered. Only 5 proteins plus members of the 2RB sequence cluster yielded peptides in the SDS fractions, and all of these had peptides from urea fractions (Fig 1, S2 Table).

Other protein families appeared in the 11 proteins found in **both** the soluble fraction and the final pellet (Fig 1). This set had 2 RR-1s, 4 RR-2s and 3 CPLCGs, and one each from CPAP3, and CPLCX. The situation in final pellet was quite different from the soluble fractions. Here the majority of recovered CPR proteins were RR-2, with a clear deficiency of RR-1, 7 recovered when 13 were expected, but the RR-2 numbers were close to the expected (S8 Table). These RR-2s include members of 3 sequence clusters that had shared peptides, pus 17 others for which we found unique peptides. There was a hint of solubility among members of the 2RB sequence cluster. The biggest difference was that the final pellet included members of 8 other CP families plus four sequences with low complexity not assigned to families (CPLCX#). It is obviously important to learn how these proteins are stabilized to understand why they were not extracted. While TWDLs, and CPFLs have been implicated in chitin-binding with the extract-renature-bind protocol [5, 8], chitin-binding is unlikely to account for their insolubility since treatment with 8M urea and boiling SDS should have freed the proteins from non-covalent attachment to chitin.

While members of the RR-1 group were more likely to be extractable than members of the RR-2 group, the difference is quantitative, not absolute. What was clear from these results was that all recovered members of the TWDL, CPLCP, CPF, and CPFL families were only found in the final pellet. This was a surprise because Andersen [14] had published a model that showed proteins that lacked chitin-binding domains fitting loosely between those proteins bound to chitin. Association between solubility and each CPR group and the other families combined using a Chi-squared contingency table test give a p = 0.0002.

#### H3—Solubility class is associated with the time in development at which mRNAs appeared

We have RT-qPCR data for most of the genes coding for CPs [4, 20, 21, 23, 24]. We display these data in S3 Table, showing levels of expression in pharate adults (P12 and P24) and in adult harvested no more than 12 h after eclosion. We also have RNA-Seq data for adult females 5 days post-eclosion [25], also summarized in S3 Table.

We anticipated that proteins found only in extracts might be those secreted immediately after eclosion, hence endocuticular. In contrast to expectation, the majority of the soluble proteins (7/11) had their highest levels of transcript at P24, a few hours prior to eclosion, while only 4 were highest right after eclosion. Nonetheless, there is a clear relation between solubility and peak time of expression (p = .0005; S8 Table). This carries through to individual solubility groups when we compared time of peak expression in the soluble fraction to that in the final pellet (p = ≤0.01; S8 Table). But proper interpretation is complicated because RR-1s had later times of expression than RR-2s, irrespective of solubility class. Thus, time of transcript presence and abundance appears to be more related to CPR class than to solubility.

The RNA-Seq data from 5 d old adult females had transcripts with a FPKM (fragments per kilobase per million mapped reads) >1 from 45 CP genes [25]. Their rank order of abundance is shown in S3 Table. The top value we found was only 5% of RpS7, a ribosomal protein. This is in sharp contrast to the relative concentration of RpS7 during periods of maximal CP synthesis. Then it was at least 2-fold lower than one-third of the CP transcripts and more than 10-fold lower for 19 genes. Eight of the 13 proteins in the soluble only category had transcripts in the mature adult females. Transcripts from far fewer were found for the other two solubility classes. Transcripts were identified for only 3 of the 11 in the set that had been recovered in both soluble and final pellet and for 10 of the 48 proteins classified as restricted to the final pellet. While these data might suggest that newly secreted proteins were the most soluble, it should be noted that once again there appears more of an association between CPR group and transcript presence than solubility category. Thus of the 19 RR-1 proteins recovered in the solubility analysis, 12 had RNA-Seq data from mature adults, whereas only 4 of the 32 RR-2s had RNA-Seq data and only 5/23 of the non CPRs (S3 Table).

#### H4—Location within the cuticle contributes to solubility

Among the proteins both extractable and in the final pellet was CPAP3-C. This is the ortholog of *gasp* (CG10287) in *Drosophila melanogaster* where it is found in the developing tracheal lumen and subsequently digested away [39, 40]. With EM immunolocalization, we found it around and within chitin-containing structures in Johnston’s organ but there it persisted into the adult stage [22]. We had speculated that its presence might determine the size of the extracellular space around the dendritic cilia, but it would not need to be removed because there was no need to clear out a space. The soluble form was completely extracted in the first three treatments. This was the only CP to have significant presence following extraction with PBS. Its biphasic solubility could be related to having both a highly soluble fraction and participating in more permanent manner in other structures. We found CPAP3-C in all 5 adult structures we had examined with LC-MS/MS (S3 Table).

Localization of other proteins is less informative. We had an antibody that should recognize both CPLCG4 and CPLCG5. It was found exclusively in endocuticle [20], but peptides from both CPLG4 and CPLCG5 were found in both gel and final pellet fractions. CPR125, which was the only RR-1 protein, among those that we tested, localized in hard cuticle and there it resided in the endocuticle. Two other final pellet proteins, CPR59/70 and CPR140 were found in both exo- and endo-cuticle. Two other antibodies, those for CPR75 and CPR12/CPR13, had been localized exclusively to soft cuticle, but of the corresponding proteins, one was found in the soluble fraction and the other in the final pellet (S3 Table).

#### H5—Solubility class is associated with presence of AAP[A/V/L] motifs

Our immunolocalization analyses revealed a subset of RR-2 proteins localized to both exo- and endo-cuticle. All of these proteins had AAP[A/V/L] motifs, while those restricted to the exocuticle did not [15]. This association does not extend to members of other CP families, for CPLCG3/4/5 found only in the endocuticle [20] lacks these motifs and CPF3 restricted to the exocuticle has 2 of them. The number of AAP[A/V/L] motifs in the proteins recovered in this analysis is shown in Fig 1, and these motifs mapped to the protein sequences are in S4 File. None of the proteins found exclusively in the soluble fractions had any, but CPR124 that yielded peptides in both soluble fractions and FP had 10. Of the proteins recovered exclusively in the final pellet, 60% had such repeats. Andersen et al. [41] suggested that when these repeats occurred at spaced intervals they might contribute to cuticle elasticity. While there was a strong association between presence of AAP[AVL] motifs and solubility class (p = 0.0030), its biological significance remains unknown and is complicated by the strong association between CP family (scored as RR-1, RR-2 and other) and number of these motifs (p = 0.0002).

#### H6—Solubility class is associated with adult structures where proteins are found

Also noteworthy about the proteins that were exclusively soluble is that 6/13 had not been found in our L-MS/MS analysis of the 5 adult structures. Of the other proteins that had been recovered, over half (32/53) were in at least 4 structures [1]. This suggests that several of the proteins that are exclusively in the soluble fractions are used in structures we had not examined. Indeed, we recently found peptides for four of them in LC-MS/MS analyses of extracts of adult palp and proboscis (Zhou, personal communication).

Zhou et al. [1] found far more proteins in both gel and final pellet samples than we had in this study (see Supplementary File 4B in that paper), but that is most likely due to incomplete extraction and carryover of what we would now classify as soluble proteins. Critical differences between the techniques used in that study and the current one are that the five structures we analyzed were homogenized, not pulverized, there was only a single SDS extraction and that was only washed with water allowing carryover of proteins that had been trapped in the pellet or not completely solubilized by SDS. More significant is that the SDS soluble fraction had 8 proteins restricted to it, 7 of which we had not recovered in this study. Their absence can be explained because all were found in only one or two of the structures with low peptide coverage, but the presence of 4 CPAP1 family members suggests that we may be premature in concluding that only the CPRs are readily soluble. Further reason for concern is that in the current data there were 2 CPAP1s (CPAP1-B2, CPAP1-M) that yielded peptides recovered in the soluble fraction, but with insufficient data to have been used (S2 Table).

#### H7—The presence of peptides with motifs implicated in chitin-binding or protein assembly is associated with solubility

We would expect to recover peptides in the chitin-binding domains of CPs if the binding did not involve covalent bonds. S1 File shows the chitin-binding domains on each CPR and CPAP sequence along with the recovered peptides. We failed to recover peptides spanning all or part of the chitin-binding domain of only 7 of the CPRs. Only 3 of these proteins were recovered exclusively in the final pellet, two had atypical Consensus regions, and peptides in their Consensus regions had been found in our earlier study [1]. The chitin-binding domains of the CPAP1 and CPAP3 families are well defined and only two members were present in this analysis: CPAP3-C, which had peptides in all three chitin-binding domains, and CPAP1-G that had none. In our earlier study, we had recovered far more members of these two families, and most, including CPAP1-G, had peptides recovered from their chitin-binding-domains. So, our conclusion in that study seem confirmed, namely that the chitin-binding domains of CPRs and CPAPs are not covalently linked to chitin. Thus for the members of these families that we recovered only in the final pellet something other than chitin-binding must have been rendering them insoluble. (See H8 for a different interpretation of these data).

#### H8—The presence of peptides with amino acids implicated in sclerotization or tyrosine cross-linking affects solubility

It might be possible to examine the peptides we recovered and gain some additional information about which amino acids might be involved in rendering proteins insoluble. Andersen [42, 43] summarized the processes involved in sclerotization and lists several amino acids that have been implicated in cross-linking of CPs based on a variety of experimental perturbations. Most often mentioned is histidine, but lysine and tyrosines have also been recovered covalently bound to sclerotizing agents and di-tyrosines have also been observed. Also implicated in cross-linking are the α-nitrogens on the amino-terminus [44, 45]. Several papers present direct experimental evidence, frequently based on detection of amino acid-catechol conjugates [46–48] or chemical elimination of the nucleophilic group from these amino acids [45]. The participation in cross-linking by the N-terminal amino acid is supported by our data. None of the recovered peptides in any fraction included the N-terminal amino acid (S2 File). But this cannot be taken as definitive evidence, for it is possible that the N-terminus was modified in various ways that prevented recognition by Mascot. Another possibility for their absence, being too long to be recovered, can be eliminated, only 4 proteins plus some in the 2RB sequence cluster had a potential first peptide longer than 40 amino acids, and we had recovered peptides as long as 55 amino acids (S2 Table).

Next to consider are histidines and lysines. If cross-linking renders proteins resistant to extraction, one might expect that the peptides recovered from proteins in the final pellet would not be ones with histidine or lysine residues. Even though we failed to find a significant relationship between histidine content and solubility (S8 Table), we did find fewer histidine residues, as both number and percent, when we compared the soluble proteins with those restricted to the final pellet (p = ≤.01; S8 Table). When there were pairs of these residues, HH or KK, peptides with them were rarely detected. Thus among the recovered proteins, we detected 40 pairs or short clusters of histidines, only six of them were in recovered peptides, and four of those were in CPLCX2. The situation with lysine pairs was not informative for about half were in recovered peptides. A homology model of RR-2 proteins [49] showed 6 histidines within the Consensus exposed on the surface where they would be accessible to participate in cross-linking. Only 17 of the 26 *An*. *gambiae* RR-2s we recovered had histidines within the consensus for a total of 34 residues. Fifteen of these were in recovered peptides (S2 File). Participation of any one of these histidine residues in protein-protein cross-linking might be sufficient to render a protein insoluble, but the peptide that participated in a cross-link on one occasion, would be free to be recovered when another molecule of the same protein had used a different histidine in a sclerotization reaction.

There is, however, a situation where histidine content might play a role and that is in distinguishing the soluble RR-1s from those recovered only from the final pellet. All but one of the soluble RR-1s had less than 2% histidine (average was 1.1%), whereas all of those in the final pellet were higher (average was 3.9%), but we failed to find a significant relationship between histidine content and solubility (S8 Table). These data are summarized in Table 2.

**Table 2:** Summary of isoelectric points and histidine, tyrosine and glutamine content of CPs in different fractions.

	Number of proteins	average pI	% histidine	% tyrosine	% glutamine
Soluble RR-1^a^	9 (10)	4.43 (4.87)	1.1	7.3	6.4
Soluble RR-2	3	5.26	1.7	4.2	10.4
Both Sol and FP RR-1	2	4.57	2.0	5.1	8.8
Both Sol and FP RR-2	4	6.32	3.6	5.4	6.6
Both Sol and FP not CPR^b^	6	7.09	5.2	8.4	2.8
Final pellet RR-1	7	6.27	3.9	7.1	11.7
Final pellet RR-2^c^	23	6.83	4.0	8.8	4.0
Final pellet not CPR	18	8.70	6.0	7.9	3.9

Isoelectric points calculated at: http://web.expasy.org/compute_pi/

^a^One RR-1 protein was an outlier and is included in the values in parentheses.

^b^Both versions of CPAP3-C were used for these calculations.

^c^Two CPRs for which peptides were recovered did not score as either RR-1 or RR-2 and are omitted here.

The *An*. *gambiae* proteome has unique proteins for 45 RR-1, 101 RR-2 and for 4 CPRs that did not score with CutProtFam-Pred (http://aias.biol.uoa.gr/CutProtFam-Pred/home.php).

The results from a statistical analysis of the data in Tables 2 and 3 are given below and in S8 Table. The soluble proteins had fewer (number and percentage) histidine residues than proteins found in the other two categories (p <0.01) but the other two categories did not differ in histidine content (p >0.05). Nor was there a significant difference in tyrosine content among the three solubility categories. There were significant differences between CP family (RR-1, RR-2, other) in pI (p < .0001), percent tyrosine (p = .0085), number of tyrosines (p = .0170), percent glutamine (p = .0054) and protein length (p = .0002). Importantly, when statistical intereaction (CP Family*solubility) was tested it was seen that only for tyrosine and glutamine content was there a significant interaction (S8 Table).

**Table 3:** Representation of CPs in final pellet analyzed against VectorBase P4.2.

Row		Batch 1	Batch 2	Batch 3
A	Protein groups recovered	139	212	203
B	Protein groups with > 1 peptide	73	127	144
C	# proteins in top 25% of B	18	32	36
D	# CPs in top 25% of B	15	20	21
E	% CPs (D/C)	83	63	58

Complete data are in S6 Table.

There were several instances where a protein recovered only in the final pellet lacked or had less than 1% histidine or tyrosine residues. It is likely that these proteins are rendered insoluble by cross-links not involving these amino acids. So, it is of interest that the four proteins in this category for histidines (CPR122, CPR140, CPR147 and CPLCX5) were all high in tyrosine residues. 10.9% when the average tyrosine content for all FP proteins was 8%. The only protein below 1% in tyrosine residues was CPLCX2 and it had 18.5% histidines, the highest for any of the recovered proteins (Fig 1).

Another postulated contributor to CP cross-linking is transglutaminase that catalyzes bonds between lysine and glutamine. Early experimental evidence for the involvement of transglutaminase was done with horseshoe crabs where incubation of RR-2 proteins with transglutaminase resulted in SDS-insoluble aggregates [50]. More recently, Shibata et al. [51] used RNAi to knock down the one copy of this enzyme in *D*. *melanogaster* and recovered several proteins from the wing that had not been identified in a 10% acetic acid extract prior to the knockdown. Among these were 2 CPs, DmelCpr97Eb and DmelCpr47Ef. When DmelCpr97Eb was knocked down with RNAi, the wings were curled; in addition, the recombinant protein bound chitin. Each of these *Drosophila* proteins has an ortholog in *An*. *gambiae*, CPR127 and CPR140, both proteins restricted to the final pellet. There were 20 instances of at least 4 glutamines (Q) occurring together in proteins. Only in CPR135 was such a stretch of glutamrtines present in a recovered peptide (S3 File). Our data revealed no significant relationship between solubility class and glutamine content.

We now appreciate that it was naïve to anticipate that we would fail to recover all peptides involved in cross-linking. This would require that the same residues were used every time a cross link was formed. Our data appear to support a situation where cross-linking occurs haphazardly via different residues in a protein, so that an assortment of peptides will be recovered, even though amino acids in them could have participated in covalent modifications. A similar conclusion about lack of binding to specific residues was reached by Hackman and Goldberg [52] in respect to non-covalent chitin-binding. They had modified histidine and tyrosine residues of fly (*Calliphora vicina*) CPs, prior to carrying out chitin-binding assays and found very little effect on the fraction that bound to chitin. They concluded:

“There is probably binding of these proteins to the chitin at many points along the chains of hydrogen bonds, apolar bonds, and ionic bonds.”

More recently, Andersen [43] reached a similar conclusion when commenting on the failure to isolate intact crosslinks between proteins from preparations treated with proteolytic enzymes:

“The difficulties are presumably cause by the complexity of the enzyme digests, since a large number of different crosslinks will be formed when several reactive compounds react more or less at random with the nucleophilic groups available in the proteins and chitin.”

#### H9—Proteins found in more than one gel slice provide evidence of cross-linking

In our first LC-MS/MS analysis of *An*. *gambiae* CPs, we commented that proteins present in more than a single gel slice might be evidence of cross-linking [12]. Only 14 CPs fell into this category. All of these proteins had also been recovered in the final pellet, lending support to the claim that the high molecular weight bands represented proteins that could patriciate in cross-linking.

In the current analysis, we had good data from10 proteins with multiple peptides present in two or more gel slices (highlighted in S4 Table). Only half of these proteins were also found in the final pellet. Six proteins (CPLCG5, CPR26, CPR62, CPR76, CPR130, CPR132) had peptides in the top slice A and lower slices. We would expect peptides from such possibly cross-linked proteins to be deficient in amino acids that could contribute to cross-links. Two proteins with sufficient data to test this are CPLCG5 and CPR26. The 8M urea fraction revealed that CPLCG5 had the same three overlapping peptides in all gel regions, and all had at least one of the potential crosslinking amino acids (H, K, Q, Y). These three peptides were also recovered from the final pellet, and are the only ones possible in a trypsin digest. The opposite was found with CPR26. Here, the same peptide was recovered from the top gel slice (A) in all the urea fractions, and it was devoid of potential cross-linking sites, but this protein was not recovered from the final pellet. The data are so sparse and our suggestion that cross-linking most likely involves different residues in the same protein indicates that the role of gel slices is probably restricted to increasing the yield of peptides and not to providing insight into protein interactions.

#### H10—The presence of peptides with GYR and YLP motifs implicated in protein-protein interactions affects solubility

Cornman [11] suggested that motifs containing GYR and YLP, common in CPs and other proteins in *D*. *melanogaster*, might play a role in protein-protein interaction. Supplementary file 1 of [11] lists the 116 protein in *An*. *gambiae* that have these sites. Among them were 20 CPs with at least one of these motifs. Eleven of these CPs were identified in our study; 6 of those were in the CPR family. The solubility data for these CPs are given in S5 Table Tab A. Ten were restricted to the final pellet, one was soluble. The soluble protein, CPR76, has peptides in all four gel slices of one of the urea extracts. Thus for all of these CPs, there was evidence compatible with cross-linking.

We also examined the other 96 *An*. *gambiae* proteins Cornman [11] had identified with one or both of these motifs. We had data for only 10; 5 had been recovered exclusively in the final pellet, 4 were soluble, one was both (S5 Table Tab B). Thus the role of these two motifs was not clarified by our data.

#### H11—Protein length is associated with solubility

It is worth noting that the soluble category had 4 proteins with fewer than 100 amino acids. None that short were found in the other two sets (Fig 1). Nonetheless, there was no significant association between solubility class and length (S8 Table).

#### H12—There is an association between isoelectric point and solubility

The major factor we examined that was significantly associated with solubility was pI (p<0.0007; S8 Table). We already knew that isoelectric points were lower for RR-1s than for RR-2 [15], but the current data show that both RR-1s and RR-2s recovered from the soluble fraction have lower isoelectric points than those recovered from either the “both” category or the final pellets (p < .01). The “both” category also had lower isoelectric points than those in the final pellet (0.05 <p<0.01). These data are summarized in Table 2 and S8 Table. The CPs from families other than CPR recovered from the final pellet, with one exception, had isoelectric points above neutrality, almost all above pH 8.

### Information gleaned from analysis using VectorBase P4.2 annotated proteins

In addition to the analyses run against a private database of CPs, we also used the entire *An*. *gambiae* proteome available at VectorBase (P4.2). This enabled us to identify all of the proteins released with the different solvents, and to learn about the abundance of CPs relative to the entire proteome. Analysis of these proteins is beyond the scope of this paper, but all of the peptides, their associated proteins, and the releasing solvents are given in S6 Table. In addition to CPs, there was good representation of muscle proteins and many proteins with multiple peptides for which no function was listed. One protein we routinely find in cuticle preparations, or those enriched in cuticle is the *An*. *gambiae* ortholog of *yellow-e* so it should be considered to be a cuticular protein. Indeed, Noh et al. [53] found that deletion of yellow-e in *Tribolium* lowered desiccation resistance.

What is important for the current study, is that the final pellet has CPs as its major components. Proteins in S7 Table are grouped by shared peptides calculated by ProteoIQ. If a protein had all unique peptides it occupies a single line in this table. Others with shared peptides were highlighted before being sorted for abundance using normalized spectral counts. The number of protein groups recovered ranged from 139 to 212, but once protein groups with only a single peptide were eliminated that number fell (Table 3). S7 Table presents the protein groups identified in the final pellets of each of the three batches and give quantitative data based on normalized spectral counts calculated by ProteoIQ. (http://www.premierbiosoft.com/protein_quantification_software/index.html). The limitations of this methods were discussed in [1]. S7 Table and Table 3 show the percent of CPs found in the top 25% of protein groups ranging from 58–83% of all well-identified proteins in the final pellet.

## Conclusions

The cuticular proteins of adult *An*. *gambiae* clearly fall into distinct solubility classes, but defining the basis for these differences remains elusive. Only CPR proteins were found in the soluble only set and most of these were in the RR-1 group. Most of the recovered CPs had transcripts prior to adult eclosion, but there was a significant relationship between time of maximum transcript abundance and solubility. Proteins in the soluble class had more transcripts at 5 d post-eclosion, but the association between presence and solubility seems to be more related to CPR group than to solubility. While EM immunolocalization data were quite limited, they revealed that even proteins localized in soft cuticle and endocuticle of hard cuticle could be restricted to the final pellet. Isoelectric point was the most consistent indicator of whether a CP extracted from pulverized adults could be solubilized or would remain in the final pellet. That property was associated with the prevalence of RR-1 proteins in the soluble fraction and the presence of CPs from other families in the final pellet. We once thought that finding abundant peptides from chitin-binding domains indicated that chitin-binding is not covalent [1]. There is, however, another possibility, namely that covalent cross-linking possibly to chitin and certainly between proteins occurs haphazardly via different residues in a protein. This is consistent with the assortment of peptides we recovered. Thus a particular amino acid in a protein may have participated in a covalent-bond in one interaction, but not in another, allowing the recovery of peptides that may sometime participate in crosslinking.

## Supporting information

S1 Photos Pictures of gels. (JPG)

S1 File Peptides found in chitin-binding domains of the CPR, CPAP1 and CPAP3 families. (DOCX)

S2 File Peptides found in CPs; histidines (H) and tyrosines (Y) are emphasized. (DOCX)

S3 File Peptides found in CPs; glutamines (Q) are emphasized. (DOCX)

S4 File Peptides found in CPs; AAP[A/V/L] motifs are emphasized. (DOCX)

S1 Table Description of samples from each extract used for LC-MS/MS analysis. (DOCX)

S2 Table Peptides recovered with each solvent by solubility class and protein names. (XLSX)

S3 Table Continuation of data in Fig 1. Solubility classes and properties of CPs.
(DOCX)

S4 Table CP peptides recovered in gel slices. (XLSX)

S5 Table CPs with Motif 1 (GYR) and Motif 2 (YLP) recognized by Cornman (ref 11). (XLSX)

S6 Table Peptides recovered using the VectorBase P4.2 database; CPs are highlighted. (XLSX)

S7 Table All proteins in the final pellet rank-ordered by normalized spectral counts. (XLSX)

S8 Table Statistical analysis of data in Fig 1 and S3 Table. (DOCX)

S9 Table Peptides and CPs in untreated and ACN extracts. (XLSX)
