# Supplementary material for: Properties of the cuticular proteins of Anopheles gambiae as revealed by serial extraction of adults
Source: PLoS One. 2017 Apr 18;12(4):e0175423. doi: 10.1371/journal.pone.0175423 (PMC5395146; doi:10.1371/journal.pone.0175423)
Supplement: S2 File — (DOCX) [file pone.0175423.s003.docx]

**S2 File Peptides found in CPs; histidines (H) and tyrosines (Y) are emphasized.**

Chitin-binding domains in CPRs and CPAP1 and CPAP3 are underlined. Peptides highlighted in green were detected in soluble fraction, in gray in final pellet, and purple in both. Peptides with amino acids in light yellow were shared among proteins. CPR names in green are RR-1, in brown RR-2, in black unclassified. Underlined names scored below threshold in CutProtFam-Pred. Arginine(R) and lysine (K) residues in the mature protein are marked to show where trypsin could cut. Complete data are in S2 Table.

**SOLUBLE ONLY**

>|CPR8|

MKQFVVFASVCCVLLAGRVVLS (signal peptide)

APAPQQGGAPAGQNPNDVQTV**RYY**SENNGLDG**YK**FT**Y**ELSDGQI**R**SEVGT**YR**DV**K**DAEG**K**DV**K**ALFVQGS**Y**SFVGPDGQT**Y**WVN**Y**TADENG**YH**P**K**VGTGPTGGIQAGQDAPVASA

>|CPR15|

MYKLFVIAALVAVAAA (signal peptide)

QNPQDAQAQVLASDSVVNPDGS**Y**N**YRY**ETSNGLAAQESGVGGQSAQGS**Y**S**Y**TGDDGVQ**Y**QVS**Y**VADENGFQPQGA**H**LPVDGPAPD**H**VL**K**TLEQI**R**ANPP**R***DDPNFSMDALNAAIA****R***LSG**KK**

(Only 1 instance of the peptide in italics with the wavy underline was detected in the final pellet. There were 12 occurrences of peptides in urea fractions.)

>|CPR23|

MKFAVVFAAVLAVALA (signal peptide)

APADE**R**DAQVL**KY**ENDNLGVDG**Y**NFQ**Y**ETSNNIN**R**AETAEL**K**NFGDDVAALVV**R**GS**Y**S**Y**TGPDGQV**Y**TVN**Y**VADENGFQPEAP**H**IP**R**L

>|CPR26|

MKMLLGAIALTLVISAFA (signal peptide)

APLDDS**R**NAEIL**RY**SSENIGIDG**YR**FEFATSDGTS**R**TEEAEL**R**NPGTDNEAIAV**R**GS**Y**S**Y**TGPDGTV**Y**VIN**Y**VADENGFQPEGA**H**IP**K**

>|CPR30|

MKHVIVLFAALAVFCAVKA (signal peptide)

QDEVQVISFNNDNNVDGS**Y**QFA**Y**EQSDGQ**KR**EE**K**GEL**K**PVEGSDEPALSV**K**GS**Y**E**Y**TDSNGQ**RYR**VD**Y**VADE**R**G**YR**PTVT**K**L

>|CPR75|

MKCEIVLLLACATLALS (signal peptide)

APQ**R**SDAEAEILQQDNNIEPDGQ**Y**Q**Y**S**Y**ETANGI**R**GQETGTL**KR**ANSPDTSDVIVAAGSIT**Y**TAPDGQVVELS**Y**TADDENGFQPAGA**H**LPTPPPIPPQIQ**K**ALD**Y**LASLPPSAN**RRR**

>|CPR76|

MRLHLSFIVLGAVLACVLG (signal peptide)

QDDG**KYR**PSP**R**ELTTQ**R**P**R**P**Y**QP**Y**VDP**RY**LQ**Y**P**Y**NT**YRY**SND**RRYY**N**R**NDG**RY**NSGNDG**KY**VP**R**NDG**RY**V**H**VDN**RYKH**VEGPSG**R**GAGQAGSAGGAGGAAGASGAGANAADTNPP**K**PATTTSAPSAPPPTPVPTLA**R**IAQV**K**VAP**K**PA**Y**APDGW**K**II**R**LENQVENDG**YHY**VFETENGILAEEAG**R**IED**K**GTAAEGL**R**SQGF**Y**Q**Y**VGDDGVV**YR**VD**Y**VADGNGFLPQGD**H**IP**K**VPPAIE**K**LL**KY**LAAQP**K**

>|CPR78|

MKVIICACVVLLLAFGGVEC (signal peptide)

APQGPATEPIPII**R**QEQEVNPDGS**Y**SWS**Y**ETGNGIVAEEQGFL**K**NPGTEQEAQVAQGE**Y**S**Y**TAPDGQLI**R**VQ**Y**IADENGFQPLGD**H**LPTPPPIPPAIQ**R**ALE**Y**LASLPPSDDAS**RRY**

>|CPR81|

MKFVVLLVAALVAATSA (signal peptide)

QI**R**PLPIPL**R**NPGV**Y**GGPEASAVILNQV**Y**EPNPDGS**Y**V**Y**S**Y**ETSNGI**R**ADQ**R**GFL**K**NPGTPGEAQVMQGS**Y**S**Y**TGPDGVV**Y**TIN**Y**IADENG**YR**AEGA**H**IPSAP**RY**NP**RY**PGQ**Y**Q

>|CPR106|

MYKQSLILLSCALALTLA (signal peptide)

APPAPLT**KR**SPQGGPDSEATVVAQDQIINEGGS**Y**A**Y**N**Y**ETSNGI**K**A**R**QTSDNGVSANGE**Y**SFLAPDGTS**Y**SVV**Y**VADENGFQPQGA**H**LPTEPPAPE**H**VI**K**LLEDL**R**ANPPSDPEFDLASLDATLA**R**L**R**ATQG

>|CPR69|

MAKILVILSVLLVLGAA (signal peptide)

APQSQQP**K**AGTPLQQ**RK**T**K**MDWEQLTNTIEEVFGITAEG**K**Q**K**EEQEASEQDEQEAADE**RKR**EFLE**R**QQE**KK**DSELEE**R**EE**R**N**R**EAVG**R**AQG**R**LPA**R**EL**HRRK**QASGQ**K**TLEG**KKK**QQQSSSQEEEQGG**KK**D**RY**FNDFPL**Y**EFS**Y**GV**H**DPATGD**KK**EQWE**KR**VGD**H**V**K**G**KY**TLDQPDGT**KR**VVE**Y**AADD**R**NGFEAVV**K**EID**R**IDE**R**N**R**GDV**R**WG**H**ADGQVAQS**Y**S**K**L**KK**VD

>|CPR128|

MSLKVLVLVGVVLSVCA (signal peptide)

VCLEAAPQAE**KY**EA**Y**E**YKY**EV**K**DPE**K**QLFFD**K**NEAGDTAG**K**VTG**RY**SVWLPDG**R**LMTI**KY**IVD**K**EGGFQPEVDFQDNANPLSG

>|CPR132|

MALKFTIFLIAAVSA (signal peptide)

ASLQD**YH**QQSQQQQ**H**QQLQQQPQLQQQQQLQQQQQQQQQQQQQQQQQQQQNDPWDL**KRY**ATDA**H**DQSDEQVG**Y**DFS**Y**SV**H**DPVTGDQ**K**SQEES**RR**NG**H**V**R**GQ**Y**SWVDADGI**R**QIVN**YR**ADD**R**TGFNAEQ**RR**EPA**HR**P**R**LN**H**ILQFIPAQTVAPL**Y**TIDTIIAPA**Y**TSVS**R**VDQI**RRR**DQTS**R**VN**R**EEISEDE**R**NG**R**EN**RH**ETV**R**ND**R**PEEP**R**ED**RR**NE**RR**NDDG**R**DE**RH**DD**RR**ED**RH**EE**RR**EE**RR**EE**RR**ED**RR**ED**RR**ED**RR**ED**RR**DTT**R**EDN**R**QSSA**R**DD**R**VDE**RH**EE**RR**TD**R**LNE**HR**EE**RR**DE**RR**EEG**R**DN**R**DD**RR**QVVQETASQSEV**R**FQAPSVS**Y**Q**Y**SN

**BOTH SOLUBLE AND FINAL PELLET**

>|CPR16|

MFRVFVIAALAAVAVA (signal peptide) QNPDADAQVLSSDSVVNPDGS**Y**QWN**Y**ETSNGI**R*AQEQGVGGQSAQGSASWTDRDGTPISLTYVADENGYQPQGDHLPR***EGPVPA**H**VL**K**TLEFI**R**ANPP**K**DDPNFNIQALEAEIA**R**LQALQ

(Peptide in bold and italics found only in soluble fraction, but it overlaps peptides present in both soluble and final pellet.)

>|CPR62|

MFSKVLILAAVTVCSVLA (signal peptide)

APQ**KR**LGGPLPLGTAESQAVILAQEQN**H**DPSGA**Y**N**YRY**ETSNGIAAQQTS**Y**DGANAAGE**Y**S**Y**TGPDGVL**YR**VA**Y**NADT**Y**GFQPQGA**H**LPVEPPVPD**H**VL**K**SLEEI**R**ANPP**R**DQEFNLAALDAQIA**R**L**R**ATLG

>|CPR10|

MASKISLIAVGLLLVNVGVNA (signal peptide)

QQ**Y**GQQLG**RR**SSQD**R**LNQL**R**S**Y**DDGA**R**QS**R**N**Y**NDL**Y**NEQ**RY**AS**R**TQDQEQQQQQQ**H**QD**RR**ESSD**Y**D**R**DD**Y**S**Y**G**Y**AV**R**DELSGDI**K**SQQEV**R**NGD**R**V**R**GQ**YR**TLESDGTE**R**IVD**Y**TADDV**R**GFNAVV**RH**QPSVGT**R**AQLV**H**TLQPAVLL**R**QPTVG**H**LVSGQ**HR**PALLTTPQQTSTVLL**R**N

>|CPR116|

MKFFVFVASLSAAVAMFGAHG (signal peptide)

AVVPVAPAVAVSTN**Y**DPLPQ**Y**T**Y**A**Y**NVQDALTGDS**K**SQQET**R**DGDIV**R**GS**Y**SLVEPDGTL**R**TVF**Y**TADPVNGFNAVVQ**R**GPLVP**K**AVVPVAAGPAILAPAPVA**R**VLG

>|CPR124|

MSLLKVAVFVALALCASA (signal peptide)

EP**K**PDPALLAAPLAAAAPLA**Y**SAP**Y**VQAPLVA**R**SFAAPAPLA**Y**SAPLAA**Y**TAPVVA**R**AA**Y**TAAAAPLA**Y**SAPVVAA**R**AAPLAAAA**Y**TAPVVAAAAPAVVAA**R**AAAPVVAAAPVVAA**R**AAPVVAAAPVQAEFTDA**Y**PQ**Y**Q**Y**A**Y**NVQDTLTGDS**K**TQEET**R**DGDIV**K**GS**Y**SLIEPDGS**RR**IVN**YY**ADPINGFNAVVQ**K**DVPVAVATPAVAVAA**K**TVVAPAVAA**K**AVVV

>|CPR130|

MKAFVLGSAVLLLASAAASG (signal peptide)

S**Y**LGVALSSQ**Y**QA**H**DGIGG**Y**S**Y**G**Y**AEPNSQ**KH**ET**K**DA**H**GIT**H**GG**Y**S**Y**VDANG**H**VQSV**KY**TADPI**H**GFQVSGTNLP**K**GPAP**H**AVPVPAWNA**Y**A**Y**APVVLG**H**NGAPLETPEVQAA**K**AA**H**FAA**H**AAA**K**A**R**L**HKR**SL**Y**APWT**Y**AAAAPVVLG**H**NGVPLDTPEVA**H**A**K**AE**H**AAA**H**A**K**ALG**H**A**Y**APAGPVPDTPEVQ**H**A**K**AA**H**LAA**H**AAA**R**AN**HH**AVAPVTTVA**H**T**HH**AV**H**AA**HY**PQ**H**VPVI**K**NGVPVETPEVQ**H**A**K**AA**H**FAAVA**K**AQG**Y**APA**H**A**H**S**YY**PQ**H**IPVI**H**NGVPVETPEVQ**H**A**K**AA**HY**AALAEASA**R**AG**H**GASWAPAG**H**EDDGS**Y**DG**R**WDN**HY**

**FINAL PELLET ONLY**

>|CPR9|

MLKLAFLVTTLVVCCHA (signal peptide)

G**RR**DVT**RHK**PQLVVVEE**Y**EE**R**QTTTLPPPP**K**P**Y**AFT**Y**SAG**R**SPG**H**VD**R**T**H**SEVSDGSGVV**R**GSFS**Y**VDP**R**NQV**R**TVE**Y**TADS**H**GF**Y**PVLS**H**LPATPQQTEAVA**R**AQE**KH**FAL**Y**A**K**IAQE**H**ADA**H**SG**R**AVEP**K**LP**K**DTVAVA**K**A**K**D**RH**FSL**Y**E**K**IAQE**H**A**R**IGAEQEAA**R**LAFEATSV**KY**EEE

>|CPR12/CPR13|

MFRFVFAAALLVATVAA (signal peptide)

GPLD**R**A**Y**S**H**QQNPDA**H**AQIVA**Y**ENVL**K**DDG**HY**NWS**Y**ETSNGIAA**H**EEGLGA**H**NANGAFS**Y**TGPDGVL**YR**VV**Y**VADENGFQPQGD**H**LPTPPPTPE**H**VF**K**TLEQI**R**ANPP**K**DQ**K**DFSLEALDATLA**R**L**R**Q**H**

>|CPR113|

MVHKTLLLCAFLGLASA (signal peptide)

A**R**LDNL**Y**GAPAPSASFQGGAGDGNLLNAP**R**QSPANQ**Y**LPPSAQGQQG**Y**PSVAPLGQQGQQQSGFNT**Y**NPQPSGPGS**Y**PGSGSAPSGPAGGSFQGTN**Y**QQTTPIPIL**RY**ENVNNGDGS**YR**FD**Y**ATGNGIQ**H**QEEGFL**R**NLGPE**K**SEQVVSGG**Y**S**Y**TAPDGQL**Y**SVQ**YK**ADANGFQPVGD**H**LPTPPPLPQALQEA**Y**DL**H**A**R**L**H**AEAAA**R**PQNPA**Y**QEPQAQ**Y**GAPQGGSQQQSS**YY**PQQPQQQQQPQ**YHH**QQQPQ**Y**QQPQQPQ**Y**NQQQTPQFSSPNAIQG**Y**PSAPANQ**Y**LPPA**KR**QQGFNPNSG**Y**T**Y**

>|CPR125|

MFRFVLLSTLLVAATA (signal peptide)

Q**Y**NGG**YHR**DP**K**TAAILSEQ**RY**QSGDG**K**FGAA**Y**TQEDGTDF**K**EETDEQGN**RR**GS**Y**S**Y**VDPTGQ**RR**TIS**Y**VAG**K**NGFQASGD**H**LPVAPPAPPQPAPQPQ**Y**QPQPQ**Y**QPQPQ**Y**NGG**R**S**Y**DDDGQ**Y**DP**R**WNDPNFSQNQ**Y**SAPAPAPAPAPV**H**N**YH**AAPVAPVAPVPQ**Y**NQ**H**N**Y**APQPQAAPAWTTTPAP**HR**FQPPG**K**LQLN**R**TPDG**Y**S**Y**TFN**K**V

>|CPR126|

MIGLKLLTVAACLAGVILA (signal peptide)

QD**Y**QEF**R**QAPL**R**IGT**K**AEEP**K**PTPVPIL**K**QIN**RH**NEDGS**Y**T**Y**G**Y**EGADGSF**K**IET**K**LATGEV**K**G**KY**G**Y**VDETG**K**V**K**VVE**Y**GAN**KY**GFQPSGEGITVPPPTLVDETTG**K**DDLLEDDDGNVPVAP**R**LQ**KHR**PAS**R**P**R**FQEVPIQQ**H**QPQQ**HH**QPQ**RHH**QQPQQQQ**HHH**QQQQPQPQ**YY**D**Y**DEQPAAPLPQP**KY**QPAPQ**Y**VQ**R**S**H**FGSAAAAAPSVGPAPP**R**PAQIPNAVPVDVV**Y**AP**K**QLPA**R**PDAE**YR**NVGPQTFPGAAPAPEP**K**I**RY**SQPAFPQAAPA**K**SANPVPLQPAFPQP**R**PQPAV**Y**QP**R**PAQG**R**STSVLDQLA**K**D**Y**ALPQGGAAPL**H**DITFG**YY**

>|CPR127|

MKSLLLLSAIVTIIAA (signal peptide)

Q**R**D**Y**TTPVPIL**K**QIN**RH**NEDGS**Y**S**Y**G**Y**EAADGTF**K**IET**KY**PNGEVQG**KY**G**Y**VDDGG**K**L**R**EIE**Y**GASN**R**GFEPQGTDINVPPPTLSNSN**Y**PPLGPNEEDDGQ**YR**EDPSI**YYK**DS**RY**NS**K**PAP**Y**NAP**R**PAPVA**Y**SPAPQQ**YYR**DAASQPEQPATS**Y**QPQ**HR**FQPQPQ**HH**QQQQQQ**YY**QQPAVPQ**HR**ADIW**H**PNA**K**VDINTGS**Y**SLS**Y**TG**R**

>|CPR151|

MMKLVVLLAVFSVIVGA (signal peptide)

Q**K**Q**H**QQQQQ**H**QQQQQQ**HH**QQQSLP**RYK**EIPIVNLENVLEVDG**K**F**RY**S**Y**EGGDGT**R**AAQDGQQIVVNNQVGTASQGQ**Y**T**Y**QGDDG**K**T**Y**SIS**Y**IADENG**YR**PVGD**H**LPTPPPVPAPIA**R**ALA**H**LA**K**LPPS**K**DGPG**RK**F

**2RA SEQUENCE CLUSTER**

>|CPR1|

MAFKFVAFAALIAVARA (signal peptide)

GLIASPAVS**Y**AAAPAVVAAAPVA**K**VA**Y**AAAPAVVAAPVA**K**VA**Y**AAQPEE**Y**DANP**HY**SFS**Y**GISDALTGDS**KSQQESRSGDVVQGSYSVVDPDGTKR**TVE**Y**TADP**H**NGFNAVV**HR**EPLAA**KTIVAAAPVATK**TIVAQPAVA**Y**AAPVA**K**TIS**Y**AAPLAT**K**TVVASPAIS**Y**AAPVA**K**LATPVA**Y**TAAL**HH**

>|CPR2/CPR4|

MAFKFVIFAAVVAVARA (signal peptide)

GLIASPAVS**Y**AAAPALVAAPVA**KVAYAAAPIAK**VA**Y**AAQPEE**Y**DANPQ**Y**SFS**Y**GISDALTGDS**KSQQESRSGDVVQGSYSVVDPDGTKRTVDYTADPHNGFNAVVRR**EPLAA**KTIVAAAPVATKVIAQPAVAYAAPVAKTISYAAPVATKTYVAQPALSYAAPLTKTYVSQPALSYAAPVAKTISYSAPLATKTYVSQPAISYAAPLATK**T**Y**VSQPALS**Y**AAPA**Y**A**YHH**

>|CPR3|

MAFKFVIFAAVVAVARA (signal peptide)

GLIASPAVS**Y**AAAPALVAAPVA**KVAYAAAPIAK**VA**Y**AAQPEE**Y**DANP**HY**SFS**Y**GISDALTGDS**KSQQESRSGDVVQGSYSVVDPDGTKRTVDYTADPHNGFNAVVRR**EPLAA**KTIVAAAPVATKVIAQPAVAYAAPVAKTISYAAPVATKTYVAQPALSYAAPLTKTYVSQPALSYAAPVAKTISYSAPLATK**T**Y**VSQPAIS**Y**AAPLA**K**T**Y**VSQPALS**Y**AAPA**Y**A**YHH**

>|CPR5|

MAFKFLTFAALVAVARA (signal peptide)

GLIASPAVS**Y**AAAPALVAAPVA**KVAYAAAPIAK**VA**Y**AAQPEE**Y**DANPQ**Y**SFS**Y**GISDALTGDS**KSQQESRSGDVVQGSYSVVDPDGTKR**TVE**Y**TADP**H**NGFNAVV**HR**EPLAA**KTIVAAAPVATKVIAQPAVAYAAPVAKTISYAAPVATKTYVAQPALSYAAPLTKTYVSQPALSYAAPVAKTISYSAPLATKTYVSQPAISYAAPLATK**T**Y**VSQPALS**Y**AAPA**Y**A**YHH**

>|CPR6|

MAFKFVVFAALVAVARA (signal peptide)

GLIASPAVS**Y**AAAPA**Y**VAAAPVA**KVAYAAAPIAK**VA**Y**AAQPEE**Y**DANPQ**Y**SFS**Y**GISDALTGDS**KSQQESRSGDVVQGSYSVVDPDGTKR**TVE**Y**TADP**H**NGFNAVV**HR**EPLAA**KTIVAAAPVATKVIAQPAVAYAAPVAK**TIS**Y**AAPVA**KTISYAAPVATK**T**Y**VAQPTLS**Y**AAPVA**K**T**Y**VSQPALS**Y**AAPA**Y**AS**YH**

**END OF SEQUENCE CLUSTER**

>|CPR58|

MVARVCATLLLALAMFGSSALA (signal peptide)

**R**PG**Y**AVD**YY**D**H**P**KY**AFN**Y**GVAD**H**STGDV**K**SQ**H**ET**R**DGDVV**K**GQ**Y**SLVEPDGSV**R**TVD**Y**TADPINGFNAVVS**K**TAPLV**H**A**H**APVV**KH**VVPAV**K**AV**HH**VPAAPVVQ**K**VV**Y**A**H**EPLL**H**VA**K**TG**HY**TE**H**APAV**HY**GD**Y**DG**YY**G**HY**DDS**HYY**

>|CPR59|

MAFFKSLVCLAVLGVAAA (signal peptide)

GVVPLATE**Y**QGD**YY**A**H**P**KY**S**Y**N**Y**GV**H**DSLTGDV**K**SQVES**R**DGDVV**K**GQ**Y**SLVEPDGSV**R**TVD**Y**TADDVNGFNAVVS**K**SAPSV**H**A**K**TVVA**H**AAPAVVA**H**AAPAV**Y**AS**H**AAPAV**Y**AS**H**AAPAV**Y**AS**H**AAPAV**Y**AAPAVVA**K**TVVS**H**AAPAV**Y**AS**H**AAPAV**Y**AS**H**AAPAV**Y**AS**H**A**Y**APA**H**GTVE**YH**G**K**AAAPLA**YYH**

>|CPR70|

MKCFVAIALLAVAVSA (signal peptide)

SPVE**Y**G**HY**AP**H**ALV**H**APVAV**H**APVL**KH**VVAEPVA**Y**P**KY**SFN**Y**GI**K**DP**H**TGDI**K**SQAEE**R**DGDVV**K**GQ**Y**SLVEPDGSV**R**TVD**Y**TADD**H**NGFNAVV**HK**SAPAVQ**K**VIAAPVA**HY**APAPVLS**HY**V**H**

**3RA SEQUENCE CLUSTER**

>|CPR83|

MAFKLCVILAIMGVANA (signal peptide)

VLLPTLV**KK**VEVEAPAE**Y**QFS**Y**SV**H**DD**H**TGDI**K**SQQEE**RH**GDDV**K**GQ**Y**TLIDADG**HRR**VVD**Y**TADE**H**NGFNAVV**RR**EPLEG**HK**LV**K**TVVPV**HK**VAVPVA**HY**VAPAL**K**VAVPVA**KY**AVPVA**K**VLAPA**H**VATVSFSAPSLT**YHY**

>|CPR84/CPR108| -- CPR84 Has an extra VPVAKVA (in bold and italics) relative to CPR108; unique peptide for that region not found.

MAFKYCILFAVVAAASA (signal peptide)

AVLPVAV**KH**IE**Y**ADAPAE**Y**QFE**Y**SV**H**DD**H**TGDI**K**SQ**H**EE**RH**GDNVVGQ**Y**TLIDADG**YRR**VVE**Y**TADE**H**NGFNAVV**RR**EPV**K**TAIPVA**K**VVAPIA**K**V**Y**AAPIA**K**VA***VPVAKVA***VPVA**K**VA**Y**PA**YHY**

**END OF SEQUENCE CLUSTER**

>|CPR110|

MFKVFVVLAAVAVGVNS (signal peptide)

VAIGVAAPATLV**K**TEE**Y**DA**H**PQ**Y**SFS**Y**DVQDSLTGDN**K**QQ**H**ET**R**DGDVVQGQ**Y**SLVEPDGT**RR**TVD**Y**TADPVNGFNAVVS**K**SADAAVV**K**TVAAAPVA**H**VA**YH**APTVAVA**H**APAVAVA**H**APVA**YH**APAATA**Y**VS**H**APA**Y**VS**H**APA**Y**VS**H**APV**Y**A**H**APVVA**H**APVLS**H**AV**YHR**

>|CPR114|

MISKVLLVASLVAASVA (signal peptide)

APV**Y**GPLS**Y**GPALS**Y**G**Y**AA**K**AIQPL**H**TVAAAPVL**H**APAIAVAAAPVL**KH**VEA**Y**DPNP**HY**SFS**Y**GVSDP**H**TGDS**KH**AEETLSNGVV**H**GS**Y**SLTEPDGTI**RK**VT**Y**TAD**K**I**H**GFNAVVE**K**SG**H**AI**H**TAPVL**KK**VVAAAPLV**H**AALP**YYHH**

**2RB SEQUENCE CLUSTER**

>|CPR115|

MAFKFAVFAAIVAVASA (signal peptide)

VAI**H**PSPLGA**YH**APLGA**YH**APLGA**YH**APLGA**YH**APLGA**Y**PAVA**KVAAPLAVAK**VAAP**Y**AD**Y**DASPQ**Y**S**Y**S**Y**AVADAVTGDN**KNQQESRSGDVVTGSYSLVEPDGTRRTVEYNADPINGFNAVVHREPLAVKAVAPIAKYAAPLAYPAVAKVAAPYAPYGYPAYGK**AILG

>|CPR117/CPR154|

MAFKFAVFAAIVAVANA (signal peptide)

VAIG**Y**PAPLGA**YH**APLGA**Y**PAVA**KVAAPVVAKVADDYDPNPQYSYSYHIADALTGDNKEQQESRSGDVVTGSYSLVEPDGTRRVVEYTADPVNGFNAVVHREPLAVKAVAPVAKIAAPLAYPAVAKVAAPYAPYGYPAYGK**AILG

>|CPR118/CPR119/CPR158|

MAFKFAVFAAIVAVASA (signal peptide)

VAI**H**PSPLGA**YH**APLGA**YH**APLGA**YH**APLGA**YH**APLGA**YH**APLGA**Y**PAVA**KVAAPLAVAKVAAPYAEYDANPQYSYSYAVADAVTGDNKNQQESRSGDVVTGSYSLVEPDGTRRTVEYNADPINGFNAVVHREPLAVKAVAPIAKYAAPLAYPAVAKVAAPYAPYGYPAYGK**AILG

>|CPR121| -- differs from CPR118/CPR119/CPR158 only in signal peptide

MVFKFAVFAAIVAVASA (signal peptide)

VAI**H**PSPLGA**YH**APLGA**YH**APLGA**YH**APLGA**YH**APLGA**YH**APLGA**Y**PAVA**KVAAPLAVAKVAAPYAEYDANPQYSYSYAVADAVTGDNKNQQESRSGDVVTGSYSLVEPDGTRRTVEYNADPINGFNAVVHREPLAVKAVAPIAKYAAPLAYPAVAKVAAPYAPYGYPAYGK**AILG

>|CPR120|

MAFKFAVFAAIVAVASA (signal peptide)

VAI**H**PSPLGA**YH**APLGA**YH**APLGA**YH**APLGA**YH**APLGA**Y**PAVA**K**IAAPLAVA**KVAAPYAEYDANPQYSYSYAVADAVTGDNKNQQESRSGDVVTGSYSLVEPDGTRRTVEYNADPINGFNAVVHREPLAVKAVAPIAKY**AAPLAFPAVA**KVAAPYAPYGYPAYGK**AILG

>|CPR122|

MALRFAVLAAFVATASA (signal peptide)

VAIG**Y**PAP**Y**GA**Y**PAVA**K**VAAPLAD**Y**DPNPQ**Y**S**Y**S**Y**AVSDALTGDN**K**SQQES**R**SGDVVSGS**Y**SLIEPDGTQ**R**VVE**Y**TADPVNGFNAVV**HR**GAGVV**KAVAPVAK**FAAPLA**Y**PAVA**K**VAAPL**Y**G

>|CPR123|

MAFKFAVFAAIVAVANA (signal peptide)

VAIG**Y**PAPLGA**Y**PAVA**KVAAPVVAKVADDYDPNPQYSYSYHIADALTGDNKEQQESRSGDVVTGSYSLVEPDGTRRVVEYTADPVNGFNAVVHREPLAVKAVAPVAKIAAPLAYPAVAK**VAAP**Y**AP**Y**G**Y**PG**Y**G**K**AILG

**END OF SEQUENCE CLUSTER**

>|CPR135|

MRAFVVATILCVGFVSA (signal peptide)

**Y**PQQAVDPA**Y**L**R**Q**YY**QQIAQAAGAQNAAQG**R**ADATPI**H**EQGAQEQQ**H**IPQ**Y**LPQGQPQ**R**TQ**Y**QQPQA**R**Q**Y**QPQ**Y**QPQQIQ**Y**VQEQQ**Y**QQPQPQL**K**VS**K**P**R**PQ**Y**LQGGQ**K**QPLEEEQED**Y**DANPS**Y**QFGFDV**K**DDEFTN**Y**QN**RK**EQ**R**DGNVI**K**GS**Y**SVVDSDGFI**R**TVT**Y**TADP**K**EGF**K**AEVS**R**QPTDIVV**K**IPTPAPQSQ**H**D**R**FASQPQSAGA**YR**VQQQPQQQQQAQP**R**P**R**PQE**Y**SQ**Y**Q

>|CPR140|

MKMFAAVLLAACLATSSLA (signal peptide)

SPAPDCPPSG**H**VSG**Y**S**Y**PAPTVQLSVG**K**AINSVSVTPGFSS**Y**SVDGEV**KY**ASVGPS**Y**SS**Y**EASPA**Y**SA**Y**GASGEDL**K**LTS**Y**EAGV**K**TVEAAPAVT**Y**TA**K**TAGVV**Y**AD**K**SPAATFASVVPSVS**Y**T**K**TVAAPAV**Y**AQPAVS**K**V**Y**TSEAAPA**Y**QVT**K**E**Y**LPPTV**K**T**Y**ATAPAVAS**Y**VSTPTVT**KY**AAAPAVSS**Y**VATAPVVS**KY**VSAGPAVS**Y**SAAPVA**K**VAT**Y**SS**Y**APAA**K**VAT**Y**ATPA**Y**G**Y**AAS**Y**APAV**K**AS**Y**AS**Y**APATS**Y**AS**Y**APATT**Y**AS**Y**TPTA**K**VAS**Y**TPAVG**Y**AA**Y**APAA**K**VA**Y**AAPAAS**Y**ASS**Y**ATTS**K**LA**Y**AAPAVT**K**TVVSAPAVAS**YY**SAPAVT**KY**SSAPAVSTA**Y**VSAPTVT**KY**AAAGVA**Y**APAVSS**Y**VAP**Y**SAQA**Y**TPAVS**KY**VSTPAVSS**YY**ATPAVS**K**VVSSPA**Y**AS**Y**VSAPAVT**KY**ASAPAVSSA**Y**VSTPVVS**K**TA**Y**SGA**Y**LAAPAVA**K**VATA**Y**GPAVAS**Y**STGPAVSA**Y**STGSA**Y**SS**Y**SVAPAVS**K**VVSSPAVAA**Y**STGPA**Y**SS**Y**SVAPAVS**K**V**Y**STPAVAA**Y**SAVPAVS**K**V**Y**STPAVAS**Y**SAVPAVS**K**V**Y**STPAVAS**Y**SASPA**Y**SS**Y**SATPAVAS**Y**SAVPAVS**K**V**Y**STPAVAS**Y**SAVPAVS**K**V**Y**STPAVAS**Y**SAVPAVS**KY**AAAAPALTTA**Y**TAPATVV**K**V**Y**SSPAVAA**Y**SAGPA**Y**SS**Y**SATPAVAA**Y**SAGPA**Y**SA**Y**SVAPAVT**KY**ATSGVAG**Y**ATSGAT**H**G**YY**AAGPAVSAA**HY**GG**YRY**AAAAPALTTA**Y**TAPATVV**K**TVAPTTVV**K**TVAE**KY**LE**HY**EDNA**RY**AFE**Y**GVNDPLTGDI**KH**QKEERDGDVV**R**GQ**Y**SLVEPDGNV**R**TVD**YY**ADWATGF**H**ATVTNS**R**DQV**H**AT**K**VLG**KR**DTV**K**A

>|CPR147|

MKIYVAVITIALIALAAT (signal peptide)

EPPAP**R**NGFTSSSSN**Y**LPPNQSFNGNNG**Y**N**Y**NSNDNG**Y**N**Y**PSSSNGEN**Y**PATSQQ**Y**GPPLGNDGNGG**Y**N**Y**EDANVQPA**KY**SFE**Y**NVQDFTSGNDFG**H**MES**R**DGD**R**TVG**RY**FVLLPDG**RK**QVVN**Y**EADQNG**YR**PTIT**Y**EDIGTGNGANSNNGA**Y**EGNGQFNG**Y**Q

>|CPR160|

MASKNPSIAWIVCVVIASLTTKGYG (signal peptide) -- atypical R&R Consensus

WNLPAS**YY**QGAGGAGAGGG**Y**FGG**R**GESTLNNQGLAGASAGSWNAGGVGGGGGFPSQGQTGAGASSGVD**R**AASFGGNQWSAGGDG**R**GLGGGVAPGGVSTGGGDLGAGGTGFQQGGTGAGVGGSGSGAPLGAGSGSGFG**Y**NGQAGGFGGSNFGQGQ**Y**AP**H**NTNGVASGASGFGGSFGGSGGN**Y**GPSG**HHH**G**YRH**PTT**Y**AG**R**PMTLV**Y**PAGGWPW**H**GVG**Y**V**YR**VPVA**H**INPDGS**Y**GFS**YY**TPNSA**R**DETG**H**ANGNVEGT**Y**GFQNDGA**KH**NFSFNAAPDVDL**R**SSIGDT**R**LGGLNPDE**Y**GPQSV**H**S**R**G**R**LPLIPATSFDGAADEQT**R**TADGLPVSVEQSTGANSWST**R**SGIDGAGSEGGSDDSSLSVVGLP**K**ATTEAGDASQPTA**R**DQVV**R**GSTE**H**SANELDG**RR**LGGFDGTATTV**R**PLGGSVEQVGA**R**PVNDVPS**Y**SNEID**R**NGVQ**Y**NGGVGIVPVD**R**S**Y**QFG**Y**QTPDAT**R**EESADQAGNV**R**GSFS**Y**NNEAG**R**NDLQ**Y**VAGTGMGF**R**PTGGSLSVPNGLPGDNGQ**R**FGPATGAGGVFGVDGG**R**SLGAGSQGAQVGGGLGGAFGGSDAGFVGDG**R**SVATGDQGAPLGGTAGSGFGMDG**R**SLAFGSQGSQLGGGVGGGFGTGVGNGFGADG**R**PLGSGTGVGVDG**R**SFGSQPGGGFGAGSGAGFGADG**R**PLGFGNQGTQTVSGTGSGAGFGADG**R**PLGFGNQGTQSGGDGVGGAFGT**R**SGSGFGAGGFGNQGASGAAFGADG**RR**LGFGNQGTQFGAGGNGASFGGSDG**R**SFGFGNQGGQGFATAD**R**GLQAATT**R**NPVT**R**SPSAGSFDGSGLSSADEGEESTTLNAPDDSEQ**R**NTFGGFGDVS**R**AGLFTNAN**RR**LVQ

>|CPR162|

MWLTLAFGLVVLVASGDA (signal peptide) -- atypical R&R Consensus

QT**RR**L**R**V**R**P**R**VLAAPSSAE**Y**VDSAEDAQQDN**R**Q**YY**AAPQ**R**AQE**R**LGDVVLVASSDED**Y**GGGQ**Y**GAPVAAA**R**P**R**ADQQQ**Y**Q**R**PAA**K**STTAAPVAA**R**Q**K**APASES**R**APPVQTI**R**N**Y**S**K**VNDDGSFTFG**Y**EAADGSF**K**EET**R**GTDCVV**R**G**KY**G**Y**IDPDGN**KR**EFT**Y**VSGNPCDPNNPDGSEEEESD**R**AEGGQEDSNENVPQN**Y**PV**RR**PVPVA**R**PTPAAPV**RHH**STPAPAP**R**PTTTVFQND**Y**QD**R**Q**R**QEQQSADAEEEVQIGQ**R**GSPP**R**PAA**R**PFAGAVTTTQ**R**P**R**VQIVSTTPSPTPTIF**H**SPAAPAAPQTVLPVNITP**K**PV**YR**VSPLPTQPTLAPTT**YR**PTSSPST**R**GPTGSIDFEAEF**KR**FQADN**K**LPSPPTPSTAPSGGAAP**K**PTGSPFG**R**PGPQLAAGNPI**Y**QSQLIFDPASGQ**Y**DTAL**Y**QQLPQSDGDFQLN**HR**IQP**Y**VAGPQQ**H**Q**HH**QPQPQPQQQQ**H**PGAGQLVTLEQLQQQSPL**YR**AQPSP**R**PATVQIPQQL**Y**Q**K**QQNELQFINSQQLFAQQLELQQSQL**R**AD**R**LEAA**KK**VTVGGPPM**HR**FQPQPQPQQQ**YY**FIQPQGPPQGAPGQIDAFL**R**G**H**NIE**Y**

>|CPR163|

MKYTLALLPLAGLITLATA (signal peptide)

Q**Y**GPAPP**R**LNIPGAIPLPPI**R**EE**R**LLPQQSPQVI**R**V**RR**PGAV**R**IAAPNAI**HH**QLPSALP**K**F**H**DIPSTVE**HK**PVTEEPEDDF**R**PAFIPQLQQP**HHHH**PSPATLASPAAPSPAASPALQFPIPADEQPSE**R**D**R**ELQQNVLS**R**FNAQEN**R**PAPIQ**R**AQIPE**R**FFATD**K**EPA**R**FPAE**R**PQPQQQQQQPTP**K**Q**YR**PAPQQIA**R**PAPIAAPQQSF**R**QQQ**H**FQDED**RR**PAP**H**QQAA**R**P**H**TQQDQD**R**Q**RK**PVAQIL**RK**W**R**EE**H**EDGSITWGFENDDGSF**K**EETIGIDCVT**R**G**RY**G**Y**VDPDGE**KR**E**Y**T**Y**ETGIQCDPNQ**R**DEDDEDNLEVD**Y**QEN**K**AVLPNGV**R**LDLNNMG**KK**QS**KR**PGGQQQQQQQQQ**YYR**N

**NON-CPRs FOUND IN BOTH SOLUBLE FRACTION AND FINAL PELLET**

>|CPAP3-C-PA| -- differences between isoforms are italicized and bolded

MKYSIVFVVALFGAAVA (signal peptide)

QESF**K**CPDDFGF**Y**P**HH**SSCD**KY**W**K**CDNNVAEL**K**TCGNGLAFDATDS**KY**LTENCD**Y**L**H**NVDCGD**R**TQLEPPISTP**H**CE**R**L**Y**GIFADAA**K**CDVFWNCWNGEAS**RY**QCSPGLA**Y**D**R**EA**R**VCMWADQVPEC**K**NEEVANGFACPAAGEISNAGSFS**RH**A**H**PEDC**RKYY**ICLEGVA**R**E**Y**GCPIGTVF**K**IGDADGTGNCEDPEDVPGCED**YY**GD***QDIKALQKKGY***

>|CPAP3-C-PC| -- differences between isoforms are italicized and bolded

MKYSIVFVVALFGAAVA (signal peptide)

QESF**K**CPDDFGF**Y**P**HH**SSCD**KY**W**K**CDNNVAEL**K**TCGNGLAFDATDS**KY**LTENCD**Y**L**H**NVDCGD**R**TQLEPPISTP**H**CE**R**L**Y**GIFADAA**K**CDVFWNCWNGEAS**RY**QCSPGLA**Y**D**R**EA**R**VCMWADQVPEC**K**NEEVANGFACPAAGEISNAGSFS**RH**A**H**PEDC**RKYY**ICLEGVA**R**E**Y**GCPIGTVF**K**IGDADGTGNCEDPEDVPGCED**YY**GD***LDLKSIRKSELLAGLALQSGGAPAATKANVKSNRPAPKDSN***

>|CPLCG4|

MKVAVVAVVLALAVVSEA (signal peptide)

GVLPWGWP**Y**AGLPAA**Y**PVAAWPPAAI**H**AA**Y**PA**Y**A**HH**GA**Y**LAAP**H**AAILAAP**H**APAASVA**HH**AGVVPGATSVTAT**R**GAV**H**VAPLPG**H**AVSQQQLNLAPAPGTI

>|CPLCG5|

MKCIVAAVAVIALAVAAEA (signal peptide)

G**Y**P**Y**AG**Y**P**Y**AG**Y**P**Y**AG**Y**GATVVQANAGAWP**Y**A**H**AA**Y**P**Y**A**H**AG**Y**P**Y**A**H**AAA**Y**PAAVAA**Y**AAP**H**AALLAAP**H**APLASVA**HH**AGVVPGATSVTAT**R**GAV**H**VAPLPG**H**AVSQ**K**QLNLAPAPGTL

>|CPLCG15|

MKCIAAVVMMAVAVAVQG (signal peptide)

TSVT**Y**WGNGAALQ**H**LAPVSVGSWVQDNS**Y**A**R**IVSPWN**Y**A**Y**QAPVATVAAAPVA**Y**AAP**Y**T**Y**QPAVAVVAQ**K**EA**RY**LAAN**R**GAV**H**EAPLPG**H**VVNQQSLNLEPAPGTL

>|CPLCX3|

MFKLVVLPLFFAAVSA (signal peptide)

G**Y**LGSPLA**Y**SAPA**Y**A**H**APLAAA**YH**AP**Y**A**Y**GAPVV**K**TVAAPVA**Y**AAPA**YH**AAPIV**K**AVAPVATS**Y**ANT**YK**VSV**K**APVA**Y**AAPAVVS**H**APVA**Y**AAPA**Y**AA**H**A**Y**AAPA**Y**AA**H**A**Y**AAPA**Y**A**H**G**YYH**

**NON-CPRS FOUND EXCLUSIVELY OR ALMOST EXCLUSIVELY ONLY IN FINAL PELLET**

>|CPAP1-G|

MEEEALVALTLQLCCGILLLCVVSG (signal peptide)

QQ**YK**QQAGSPASSASSAASSSSDAVPAAANNVA**YR**A**R**P**Y**SNQ**Y**SSSSSEEEEDD**R**PVAS**Y**NSG**R**QQQQGNTL**KK**SF**KK**PS**Y**SSEELEQEEEPD**R**LTLLLE**K**SQFQCTG**R**TTG**YY**ADESLGCEVF**HY**CQENQ**KH**SWICPEGFTF**H**QV**H**LICMPPSGDNICEQSS**KYH**FVND**Y**L**YK**PINMEE**H**MT**K**PNVTL**RY**SE**RYY**PENF**Y**VDE**RHY**DEE**R**VL**R**Q**H**EE**RH**QPQQPI**K**QT**YHH**QPQQQTI**RK**QPV**Y**ATTPSS**YR**LPSSPQPT**H**SV**YR**SPDEINISLQQ**RR**PAAQPGS**Y**IQSTTP**RY**EDESE**Y**DS**Y**E**RK**

>|CPF2|

MAFKFVVFLASLAVASA (signal peptide)

G**Y**LEAD**H**AVQ**Y**AAPVA**HY**SPASAVS**Y**STISQAAPA**K**LA**Y**AAPVA**K**TVS**Y**AAPQV**Y**AAPQV**Y**AAAPVT**K**T**Y**VSSPAVGAT**H**ESTI**R**S**H**DSTIS**HY**S**K**AVDTP**Y**SSV**RK**SDT**R**ITNELP**KY**A**Y**AQPVLA**K**QVA**Y**AAAPAV**H**TT**Y**AAPAAV**H**TS**Y**A**H**AAPAVT**Y**A**H**AAPA**Y**QT**Y**A**H**AAPAAV**H**TS**Y**T**H**AAPAV**H**AT**Y**AAPAVQT**Y**A**H**AAPAV**H**TT**Y**AAPAV**H**TS**Y**A**H**AAPAV**H**AT**Y**AAPAAVQT**Y**A**H**AAPAV**H**TST**K**TLT**Y**SPAVQVA**H**TT**Y**EDA**H**A**HY**AW

>|CPF3|

MYRFVALFALVAVSQA (signal peptide)

A**Y**TLNPAGPT**Y**AGI**H**TPAITSQQSNIL**R**S**Y**GNLGQIST**Y**S**K**TIDTP**Y**SSVS**K**SDV**R**VSNPGLAVG**H**IAAS**Y**P**H**PIAAPA**Y**G**H**VG**Y**AATAL**K**NPALLGVA**Y**SAAPAVA**H**MT**Y**SNGLGIN**Y**AW

>|CPF4|

MAFKFVILAALVAAVSA (signal peptide)

GGPAA**Y**SIAAPSADF**H**SVGAS**H**E**H**TV**K**GL**Y**GQNVLSQ**Y**S**K**AVDSA**H**SSV**R**V**H**SS**R**LSNDG**Y**A**Y**AAPAV**KY**AAPA**Y**AA**HY**AAPAV**HY**PAAA**HY**AAPAV**HY**PAAA**HY**AAPAV**HY**AA**H**APIV**K**AA**Y**PAA**Y**AAPLA**YK**TPLAAPVAAV**H**GGSVVQFAGLGAS**Y**AW

>|CPFL1|

MAFKIVVLFATLACASA (signal peptide)

G**Y**VEPE**HHH**LS**Y**AAAPVA**HY**SSAPAVS**Y**SSIT**RH**ETP**K**VAVA**K**QVT**Y**AEPAV**HY**AAPLT**K**T**Y**AV**H**EPAL**K**TVVAQPA**Y**T**K**TV**Y**AQEPA**H**V**Y**A**H**AAPVVAA**K**TVS**Y**AAPQV**HY**QAAPQV**HY**QAAPALV**K**NVE**Y**T**K**TLA**Y**APVT**K**TLVSEPT**Y**T**KH**VVAEPT**Y**T**K**TLLAQPA**Y**T**KY**VSQPT**Y**T**K**TLVAE**H**QPL**YHH**QPAV**Y**A**H**AAPVVAA**K**TVS**Y**AAPAA**H**VS**H**VS**Y**ADNAA**HY**AW

>|CPLCA1|

MFRLVVLSVVLAVAAA (signal peptide)

APGA**H**LV**H**SAPLA**Y**STVVAAAPALVAQ**K**EIS**Y**Q**K**SIVEEPTVA**H**VGTIE**K**SVPTG**Y**S**H**QSFTQ**YH**N**K**QVAEPVFAPAV**KKTVVSTPVEKTTYVQAAAPVVHAAPAVYAAPVQTVYAAAPVAKTYAYAAPVEKTYTYAAAPAAISYEAAPVAYAAPLK**TS**Y**VSS**Y**PSV**Y**AAPAV**Y**A**H**D**Y**

Most peptides were shared with CPLCA2 that had no unique peptides.

>|CPLCA3|

MLKLVVLSAVLAVVAA (signal peptide)

**R**PGALT**Y**SAPLA**Y**APATLIA**K**PEI**YY**Q**K**SIIEEPTVA**H**VGSLV**K**TIPTAVS**H**QSSTVV**H**NSA**K**ITEPI**Y**APAV**K**QTLVSTPIA**K**TT**Y**FAAPAA**Y**A**Y**AAPALA**YH**DA**Y**A**YHH**L

>|CPLCG14|

MKCIIVAVIVALAVAAEG (signal peptide)

**Y**GVSS**Y**AVPLA**Y**SVPQTTVVQQNVAP**KY**VVSG**Y**AAP**Y**VASP**Y**VAAA**H**S**Y**AVPAAVS**Y**ASTAV**H**AVPAVT**Y**AAP**H**ATV**Y**AAPVQQEA**RY**VAAN**R**GAV**H**EAPLAG**H**AVNQQSLNLAPAPGTL

>|CPLCP8|

MKGFVVFVMAIALVAS (signal peptide)

AEI**KKK**DAEAPAEAEANGE**KK**QE**KR**GLWDLG**Y**G**Y**ES**H**GWDS**HK**S**H**GWEEP**H**VTTIT**KK**V**H**VP**Y**PVEVE**KH**VP**Y**PV**K**VP**Y**PVTVE**KH**VPVVVE**KK**VPV**Y**VE**KH**VPV**H**VD**R**PVP**Y**PV**K**VPV**K**VV**HK**E**Y**VEVP**K**P**Y**PV**H**VE**KH**VPVVV**KK**PV**Y**VE**KH**VPVVV**K**S**H**GWEP**H**S**H**S**Y**SEF**H**SW

>|CPLCP12|

MKFFICLSALLLVSANA (signal peptide)

ESEQ**KK**SAADS**K**AAVPLE**KK**LD**KR**GLLSLG**Y**G**Y**GINGLDVG**Y**IGGG**H**LGGA**YH**EA**H**D**HHY**G**H**GV**Y**LGG**H**TDVT**K**TVTLV**K**GVPVP**Y**TVE**KH**VP**Y**TVE**KH**VP**Y**PV**K**VPVPQP**Y**EVV**KH**VPV**H**V**K**E**Y**V**K**VPV**H**VPQP**Y**PVE**KK**VP**Y**PV**H**VPVD**R**P**Y**PV**K**VFVPQP**Y**EVT**KH**VP**Y**PV**K**VPVPQP**Y**EVT**KH**VPVPV**K**VEVPVPVP**Y**TVE**KK**VP**Y**PV**K**VPVD**R**P**Y**PV**H**VPAP**Y**PVEVE**K**PVP**Y**TVE**K**PVP**Y**EV**K**VPVD**R**P**Y**PVPVE**K**PVP**Y**PV**K**VPVP**K**P**YY**VE**KH**IP**Y**TVE**K**PVPVPV**K**VPID**R**P**Y**PVTVE**KH**IPVE**K**PVPVPV**K**VPVAVPVPV**HH**D**HHHHH**LE**H**L**H**EP**HHHHHH**DVS**Y**TSFSG**Y**GQD**Y**S**YHH**

>|CPLCX2|

MYKLFVVACFLAVAAA (signal peptide)

APGVVLPA**H**TVV**H**SAPLV**H**AAPVAVS**H**SSS**H**VV**HH**APVV**K**TVAV**H**SAPVVAV**HH**APVV**K**ALPVV**HH**APVV**HH**APVL**K**TVV**H**SAPVV**H**SV**H**PVPVV**K**SV**H**VASPVLV**HH**

>|CPLCX4|

MKILLLLGLIAAVRA (signal peptide)

APG**YY**GD**H**GLS**Y**VAAAPAP**Y**V**KY**AAPAVSVV**H**AAPAPVL**KY**AVAPAPIV**K**AVAPAATS**Y**ATI**H**QV**H**APVV**H**AAPVV**KY**AAPAPVVS**Y**V**H**SAPAVVA**H**APAPLL**KY**APA**YH**GW

>|CPLCX5|

MFTKLICIAALAISCASA (signal peptide)

**K**PGLLAPVAAPVA**Y**AAGPAVVTAQSSQVVA**R**N**Y**NGIAPLA**Y**TAPAVA**Y**AAPAVA**K**VAAPLA**Y**AAPAPLAAAA**Y**AAAPLAAP**Y**VAP**Y**AAP**Y**AAP**Y**AAA**Y**AAP**Y**AAA**Y**AAP**Y**A**K**AFASPLLA**R**AP**Y**QLW

>|CPLCX10| (AGAP006970)

MLKKTIVLSCLVAVVLC (signal)

AADLETAETSWGGGGGGGWSSGGGGGGWSSGGGGG**Y**GGG**KK**VIIISSGGGG**H**GGSGGWSSGG**R**SLGGGGGWSSGGSGLG**K**GWSSGGGS**Y**GGGGS**Y**GGGG**Y**S**K**GWPVS**K**GWSSGGSSFGGG**Y**GGGSSFGGG**H**SGGGWSSGGSSLGGWPSGGGVS**K**GWPSSGGG**Y**GGSQGWSSGGG**Y**GGGS**H**GGWSSGGSGLGGG**Y**GGGGGGWSSG**R**SLGGSGGWSSGGLSS**K**GWPSSGGS**K**GWPSGG**Y**GGGSSGWSSGGSGWSGSSGW

>|TWDL1|

MKTFVLVSCCLVLASA (signal peptide)

**R**PEAG**Y**S**Y**N**R**PSTGGSFGGGSQQAIIQ**KH**I**Y**V**H**VPPPEPEEV**R**VQ**R**PIQLAAPQ**KHYK**IIFI**K**APSAPS**Y**QAPQIPIQPQNEE**K**TLV**Y**VLV**KK**PDEQQDIVIPTPAPTQPS**K**PEV**Y**FI**KYK**TQ**K**ESSGGAASGG**Y**ASAGGFGGDLGGGLGGGDLGG**H**GSLVGGDLGG**H**GG**H**GG**H**GGDLGG**H**GGSGASAPAAQ**Y**GPPG**K**SGP**Y**

>|TWDL9|

MKVLVVLACVAIVVA (signal peptide)

**R**PEPPVGG**Y**S**HHHH**GG**H**GG**H**SG**Y**N**Y**NAPVPA**H**TQPFG**H**QAQFSGVAQSAGGSFSDTLSGAVTGSFSGIANTLSAGNTFTNANSFSNANAFTQSNANAFTQSNANAFSNAGSFVAPQQQIVQ**KH**I**Y**V**H**VPPPEPQQSFQQQIVAPGL**R**Q**KHYK**IIFI**K**TP**H**QQPSAAQLALQQSQTEE**K**TIV**Y**VLV**KK**PEAVGDISLPLPAVT**K**PS**K**PEV**Y**FI**KYR**TNTEEVQAPVGLNVGSSATAQADAGGLGFLSSSGAAQSSAVAQSAAVAQSSAVAGA**H**S**H**SNVPAQQ**Y**GTPA**HHH**GG**YH**

>|TWDL11|

MKVFVVLSVVLACAAA (signal peptide)

**R**PEAGFSS**Y**SSAPSFTSVGDFGGP**Y**SGSSSGGSSG**Y**N**Y**NPAPQIVQ**KH**I**Y**V**H**VPPPE**K**EEV**HY**P**R**VSPVAPAQ**KHYK**IIFI**K**APSPPAP**K**APIIPVQPQNEE**K**TLV**Y**VL**HKK**PEEPQDIVIPTPPPT**K**PS**K**PEV**Y**FI**KYK**TQ**K**EQSQP**K**PE**Y**GPPGQSGP**Y**

>|TWDL12|

MKSLLILFACIVCALA (signal peptide)

**R**PEPEPP**R**A**R**IVVPA**K**QQQLPQ**Y**E**Y**GAP**K**PE**Y**GPPAEE**Y**GPPPPTV**Y**GPPA**R**E**Y**GPPP**K**LIT**K**NV**Y**V**H**VPPEEPTEIV**K**SPVLEAPIP**KKHYK**IIFI**K**APAPPAPI**K**QVIPPQPQDE**HK**TLV**Y**VLV**KK**PEDPAPLEIPVPETTEPN**K**PEV**Y**FI**KYK**EGE**K**EP**HK**Q**Y**GPPAPA**Y**GPPSGPA**RY**QQF
