# Supplementary material for: Properties of the cuticular proteins of Anopheles gambiae as revealed by serial extraction of adults
Source: PLoS One. 2017 Apr 18;12(4):e0175423. doi: 10.1371/journal.pone.0175423 (PMC5395146; doi:10.1371/journal.pone.0175423)
Supplement: S4 File — (DOCX) [file pone.0175423.s005.docx]

**S4 File Peptides found in CPs; AAP[A/V/L] motifs are emphasized.**

AAP[AVL] motifs are shown in purple, font 14, and bold type. Chitin-binding domains in CPRs and CPAP1 and CPAP3 are underlined. Peptides highlighted in green were detected in soluble fraction, in gray in final pellet, and purple in both. Peptides with amino acids in light yellow were shared among proteins. CPR names in green are RR-1, in brown RR-2, in black unclassified. Underlined names scored below threshold in CutProtFam-Pred. Arginine (R) and lysine (K) residues in the mature protein are marked to show where trypsin could cut. Complete data are in S2 Table.

**SOLUBLE ONLY**

>|CPR8|

MKQFVVFASVCCVLLAGRVVLS (signal peptide)

APAPQQGGAPAGQNPNDVQTV**R**YYSENNGLDGY**K**FTYELSDGQI**R**SEVGTY**R**DV**K**DAEG**K**DV**K**ALFVQGSYSFVGPDGQTYWVNYTADENGYHP**K**VGTGPTGGIQAGQDAPVASA

>|CPR15|

MYKLFVIAALVAVAAA (signal peptide)

QNPQDAQAQVLASDSVVNPDGSYNY**R**YETSNGLAAQESGVGGQSAQGSYSYTGDDGVQYQVSYVADENGFQPQGAHLPVDGPAPDHVL**K**TLEQI**R**ANPP**R*DDPNFSMDALNAAIAR***LSG**KK**

(Only 1 instance of the peptide in bold and italics underline was detected in the final pellet. There were 12 occurrences of peptides in urea fractions.)

>|CPR23|

MKFAVVFAAVLAVALA (signal peptide)

APADE**R**DAQVL**K**YENDNLGVDGYNFQYETSNNIN**R**AETAEL**K**NFGDDVAALVV**R**GSYSYTGPDGQVYTVNYVADENGFQPEAPHIP**R**L

>|CPR26|

MKMLLGAIALTLVISAFA (signal peptide)

APLDDS**R**NAEIL**R**YSSENIGIDGY**R**FEFATSDGTS**R**TEEAEL**R**NPGTDNEAIAV**R**GSYSYTGPDGTVYVINYVADENGFQPEGAHIP**K**

>|CPR30|

MKHVIVLFAALAVFCAVKA (signal peptide)

QDEVQVISFNNDNNVDGSYQFAYEQSDGQ**KR**EE**K**GEL**K**PVEGSDEPALSV**K**GSYEYTDSNGQ**R**Y**R**VDYVADE**R**GY**R**PTVT**K**L

>|CPR75|

MKCEIVLLLACATLALS (signal peptide)

APQ**R**SDAEAEILQQDNNIEPDGQYQYSYETANGI**R**GQETGTL**KR**ANSPDTSDVIVAAGSITYTAPDGQVVELSYTADDENGFQPAGAHLPTPPPIPPQIQ**K**ALDYLASLPPSAN**RRR**

>|CPR76|

MRLHLSFIVLGAVLACVLG (signal peptide)

QDDG**K**Y**R**PSP**R**ELTTQ**R**P**R**PYQPYVDP**R**YLQYPYNTY**R**YSND**RR**YYN**R**NDG**R**YNSGNDG**K**YVP**R**NDG**R**YVHVDN**R**Y**K**HVEGPSG**R**GAGQAGSAGGAGGAAGASGAGANAADTNPP**K**PATTTSAPSAPPPTPVPTLA**R**IAQV**K**VAP**K**PAYAPDGW**K**II**R**LENQVENDGYHYVFETENGILAEEAG**R**IED**K**GTAAEGL**R**SQGFYQYVGDDGVVY**R**VDYVADGNGFLPQGDHIP**K**VPPAIE**K**LL**K**YLAAQP**K**

>|CPR78|

MKVIICACVVLLLAFGGVEC (signal peptide)

APQGPATEPIPII**R**QEQEVNPDGSYSWSYETGNGIVAEEQGFL**K**NPGTEQEAQVAQGEYSYTAPDGQLI**R**VQYIADENGFQPLGDHLPTPPPIPPAIQ**R**ALEYLASLPPSDDAS**RR**Y

>|CPR81|

MKFVVLLVAALVAATSA (signal peptide)

QI**R**PLPIPL**R**NPGVYGGPEASAVILNQVYEPNPDGSYVYSYETSNGI**R**ADQ**R**GFL**K**NPGTPGEAQVMQGSYSYTGPDGVVYTINYIADENGY**R**AEGAHIPSAP**R**YNP**R**YPGQYQ

>|CPR106|

MYKQSLILLSCALALTLA (signal peptide)

APPAPLT**KR**SPQGGPDSEATVVAQDQIINEGGSYAYNYETSNGI**K**A**R**QTSDNGVSANGEYSFLAPDGTSYSVVYVADENGFQPQGAHLPTEPPAPEHVI**K**LLEDL**R**ANPPSDPEFDLASLDATLA**R**L**R**ATQG

>|CPR69|

MAKILVILSVLLVLGAA (signal peptide)

APQSQQP**K**AGTPLQQ**RK**T**K**MDWEQLTNTIEEVFGITAEG**K**Q**K**EEQEASEQDEQEAADE**RKR**EFLE**R**QQE**KK**DSELEE**R**EE**R**N**R**EAVG**R**AQG**R**LPA**R**ELH**RRK**QASGQ**K**TLEG**KKK**QQQSSSQEEEQGG**KK**D**R**YFNDFPLYEFSYGVHDPATGD**KK**EQWE**KR**VGDHV**K**G**K**YTLDQPDGT**KR**VVEYAADD**R**NGFEAVV**K**EID**R**IDE**R**N**R**GDV**R**WGHADGQVAQSYS**K**L**KK**VD

>|CPR128|

MSLKVLVLVGVVLSVCA (signal peptide)

VCLEAAPQAE**K**YEAYEY**K**YEV**K**DPE**K**QLFFD**K**NEAGDTAG**K**VTG**R**YSVWLPDG**R**LMTI**K**YIVD**K**EGGFQPEVDFQDNANPLSG

>|CPR132|

MALKFTIFLIAAVSA (signal peptide)

ASLQDYHQQSQQQQHQQLQQQPQLQQQQQLQQQQQQQQQQQQQQQQQQQQNDPWDL**KR**YATDAHDQSDEQVGYDFSYSVHDPVTGDQ**K**SQEES**RR**NGHV**R**GQYSWVDADGI**R**QIVNY**R**ADD**R**TGFNAEQ**RR**EPAH**R**P**R**LNHILQFIPAQTVAPLYTIDTIIAPAYTSVS**R**VDQI**RRR**DQTS**R**VN**R**EEISEDE**R**NG**R**EN**R**HETV**R**ND**R**PEEP**R**ED**RR**NE**RR**NDDG**R**DE**R**HDD**RR**ED**R**HEE**RR**EE**RR**EE**RR**ED**RR**ED**RR**ED**RR**ED**RR**DTT**R**EDN**R**QSSA**R**DD**R**VDE**R**HEE**RR**TD**R**LNEH**R**EE**RR**DE**RR**EEG**R**DN**R**DD**RR**QVVQETASQSEV**R**FQAPSVSYQYSN

**BOTH SOLUBLE AND FINAL PELLET**

>|CPR16|

MFRVFVIAALAAVAVA (signal peptide) QNPDADAQVLSSDSVVNPDGSYQWNYETSNGI**R*AQEQGVGGQSAQGSASWTDRDGTPISLTYVADENGYQPQGDHLPR***EGPVPAHVL**K**TLEFI**R**ANPP**K**DDPNFNIQALEAEIA**R**LQALQ

(Peptide in bold and italics found only in soluble fraction, but it overlaps shorter peptides present in both soluble and final pellet.)

>|CPR62|

MFSKVLILAAVTVCSVLA (signal peptide)

APQ**KR**LGGPLPLGTAESQAVILAQEQNHDPSGAYNY**R**YETSNGIAAQQTSYDGANAAGEYSYTGPDGVLY**R**VAYNADTYGFQPQGAHLPVEPPVPDHVL**K**SLEEI**R**ANPP**R**DQEFNLAALDAQIA**R**L**R**ATLG

>|CPR10|

MASKISLIAVGLLLVNVGVNA (signal peptide)

QQYGQQLG**RR**SSQD**R**LNQL**R**SYDDGA**R**QS**R**NYNDLYNEQ**R**YAS**R**TQDQEQQQQQQHQD**RR**ESSDYD**R**DDYSYGYAV**R**DELSGDI**K**SQQEV**R**NGD**R**V**R**GQY**R**TLESDGTE**R**IVDYTADDV**R**GFNAVV**R**HQPSVGT**R**AQLVHTLQPAVLL**R**QPTVGHLVSGQH**R**PALLTTPQQTSTVLL**R**N

>|CPR116|

MKFFVFVASLSAAVAMFGAHG (signal peptide)

AVVPVAPAVAVSTNYDPLPQYTYAYNVQDALTGDS**K**SQQET**R**DGDIV**R**GSYSLVEPDGTL**R**TVFYTADPVNGFNAVVQ**R**GPLVP**K**AVVPVAAGPAILAPAPVA**R**VLG

>|CPR124|

MSLLKVAVFVALALCASA (signal peptide)

EP**K**PDPALL**AAPL**AA**AAPL**AYSAPYVQAPLVA**R**SF**AAPA**PLAYSAPLAAYTAPVVA**R**AAYTAA**AAPL**AYSAPVVAA**RAAPL**AAAAYTAPVVAA**AAPA**VVAA**R**A**AAPV**VA**AAPV**VAA**RAAPV**VA**AAPV**QAEFTDAYPQYQYAYNVQDTLTGDS**K**TQEET**R**DGDIV**K**GSYSLIEPDGS**RR**IVNYYADPINGFNAVVQ**K**DVPVAVATPAVAVAA**K**TVVAPAVAA**K**AVVV

>|CPR130|

MKAFVLGSAVLLLASAAASG (signal peptide)

SYLGVALSSQYQAHDGIGGYSYGYAEPNSQ**K**HET**K**DAHGITHGGYSYVDANGHVQSV**K**YTADPIHGFQVSGTNLP**K**GPAPHAVPVPAWNAYAYAPVVLGHNGAPLETPEVQAA**K**AAHFAAHAAA**K**A**R**LH**KR**SLYAPWTYAA**AAPV**VLGHNGVPLDTPEVAHA**K**AEHAAAHA**K**ALGHAYAPAGPVPDTPEVQHA**K**AAHLAAHAAA**R**ANHHAVAPVTTVAHTHHAVHAAHYPQHVPVI**K**NGVPVETPEVQHA**K**AAHFAAVA**K**AQGYAPAHAHSYYPQHIPVIHNGVPVETPEVQHA**K**AAHYAALAEASA**R**AGHGASWAPAGHEDDGSYDG**R**WDNHY

**FINAL PELLET ONLY**

>|CPR9|

MLKLAFLVTTLVVCCHA (signal peptide)

G**RR**DVT**R**H**K**PQLVVVEEYEE**R**QTTTLPPPP**K**PYAFTYSAG**R**SPGHVD**R**THSEVSDGSGVV**R**GSFSYVDP**R**NQV**R**TVEYTADSHGFYPVLSHLPATPQQTEAVA**R**AQE**K**HFALYA**K**IAQEHADAHSG**R**AVEP**K**LP**K**DTVAVA**K**A**K**D**R**HFSLYE**K**IAQEHA**R**IGAEQEAA**R**LAFEATSV**K**YEEE

>|CPR12/CPR13|

MFRFVFAAALLVATVAA (signal peptide)

GPLD**R**AYSHQQNPDAHAQIVAYENVL**K**DDGHYNWSYETSNGIAAHEEGLGAHNANGAFSYTGPDGVLY**R**VVYVADENGFQPQGDHLPTPPPTPEHVF**K**TLEQI**R**ANPP**K**DQ**K**DFSLEALDATLA**R**L**R**QH

>|CPR113|

MVHKTLLLCAFLGLASA (signal peptide)

A**R**LDNLYGAPAPSASFQGGAGDGNLLNAP**R**QSPANQYLPPSAQGQQGYPSVAPLGQQGQQQSGFNTYNPQPSGPGSYPGSGSAPSGPAGGSFQGTNYQQTTPIPIL**R**YENVNNGDGSY**R**FDYATGNGIQHQEEGFL**R**NLGPE**K**SEQVVSGGYSYTAPDGQLYSVQY**K**ADANGFQPVGDHLPTPPPLPQALQEAYDLHA**R**LHAEAAA**R**PQNPAYQEPQAQYGAPQGGSQQQSSYYPQQPQQQQQPQYHHQQQPQYQQPQQPQYNQQQTPQFSSPNAIQGYPSAPANQYLPPA**KR**QQGFNPNSGYTY

>|CPR125|

MFRFVLLSTLLVAATA (signal peptide)

QYNGGYH**R**DP**K**TAAILSEQ**R**YQSGDG**K**FGAAYTQEDGTDF**K**EETDEQGN**RR**GSYSYVDPTGQ**RR**TISYVAG**K**NGFQASGDHLPVAPPAPPQPAPQPQYQPQPQYQPQPQYNGG**R**SYDDDGQYDP**R**WNDPNFSQNQYSAPAPAPAPAPVHNYH**AAPV**APVAPVPQYNQHNYAPQPQ**AAPA**WTTTPAPH**R**FQPPG**K**LQLN**R**TPDGYSYTFN**K**V

>|CPR126|

MIGLKLLTVAACLAGVILA (signal peptide)

QDYQEF**R**QAPL**R**IGT**K**AEEP**K**PTPVPIL**K**QIN**R**HNEDGSYTYGYEGADGSF**K**IET**K**LATGEV**K**G**K**YGYVDETG**K**V**K**VVEYGAN**K**YGFQPSGEGITVPPPTLVDETTG**K**DDLLEDDDGNVPVAP**R**LQ**K**H**R**PAS**R**P**R**FQEVPIQQHQPQQHHQPQ**R**HHQQPQQQQHHHQQQQPQPQYYDYDEQP**AAPL**PQP**K**YQPAPQYVQ**R**SHFGSAAAAAPSVGPAPP**R**PAQIPNAVPVDVVYAP**K**QLPA**R**PDAEY**R**NVGPQTFPG**AAPA**PEP**K**I**R**YSQPAFPQ**AAPAK**SANPVPLQPAFPQP**R**PQPAVYQP**R**PAQG**R**STSVLDQLA**K**DYALPQGG**AAPL**HDITFGYY

>|CPR127|

MKSLLLLSAIVTIIAA (signal peptide)

Q**R**DYTTPVPIL**K**QIN**R**HNEDGSYSYGYEAADGTF**K**IET**K**YPNGEVQG**K**YGYVDDGG**K**L**R**EIEYGASN**R**GFEPQGTDINVPPPTLSNSNYPPLGPNEEDDGQY**R**EDPSIYY**K**DS**R**YNS**K**PAPYNAP**R**PAPVAYSPAPQQYY**R**DAASQPEQPATSYQPQH**R**FQPQPQHHQQQQQQYYQQPAVPQH**R**ADIWHPNA**K**VDINTGSYSLSYTG**R**

>|CPR151|

MMKLVVLLAVFSVIVGA (signal peptide)

Q**K**QHQQQQQHQQQQQQHHQQQSLP**R**Y**K**EIPIVNLENVLEVDG**K**F**R**YSYEGGDGT**R**AAQDGQQIVVNNQVGTASQGQYTYQGDDG**K**TYSISYIADENGY**R**PVGDHLPTPPPVPAPIA**R**ALAHLA**K**LPPS**K**DGPG**RK**F

**2RA SEQUENCE CLUSTER**

>|CPR1|

MAFKFVAFAALIAVARA (signal peptide)

GLIASPAVSYA**AAPA**VVA**AAPV**A**K**VAYA**AAPA**VV**AAPV**A**K**VAYAAQPEEYDANPHYSFSYGISDALTGDS**KSQQESRSGDVVQGSYSVVDPDGTKR**TVEYTADPHNGFNAVVH**R**EPLAA**KTIVAAAPVATK**TIVAQPAVAY**AAPV**A**K**TISY**AAPL**AT**K**TVVASPAISY**AAPV**A**K**LATPVAYTAALHH

>|CPR2/CPR4|

MAFKFVIFAAVVAVARA (signal peptide)

GLIASPAVSYA**AAPA**LV**AAPV**A**KVAYAAAPIAK**VAYAAQPEEYDANPQYSFSYGISDALTGDS**KSQQESRSGDVVQGSYSVVDPDGTKRTVDYTADPHNGFNAVVRR**EPLAA**KTIVAAAPVATKVIAQPAVAYAAPVAKTISYAAPVATKTYVAQPALSYAAPLTKTYVSQPALSYAAPVAKTISYSAPLATKTYVSQPAISYAAPLATK**TYVSQPALSY**AAPA**YAYHH

>|CPR3|

MAFKFVIFAAVVAVARA (signal peptide)

GLIASPAVSYA**AAPA**LV**AAPV**A**KVAYAAAPIAK**VAYAAQPEEYDANPHYSFSYGISDALTGDS**KSQQESRSGDVVQGSYSVVDPDGTKRTVDYTADPHNGFNAVVRR**EPLAA**KTIVAAAPVATKVIAQPAVAYAAPVAKTISYAAPVATKTYVAQPALSYAAPLTKTYVSQPALSYAAPVAKTISYSAPLATK**TYVSQPAISY**AAPL**A**K**TYVSQPALSY**AAPA**YAYHH

>|CPR5|

MAFKFLTFAALVAVARA (signal peptide)

GLIASPAVSYA**AAPA**LV**AAPV**A**KVAYAAAPIAK**VAYAAQPEEYDANPQYSFSYGISDALTGDS**KSQQESRSGDVVQGSYSVVDPDGTKR**TVEYTADPHNGFNAVVH**R**EPLAA**KTIVAAAPVATKVIAQPAVAYAAPVAKTISYAAPVATKTYVAQPALSYAAPLTKTYVSQPALSYAAPVAKTISYSAPLATKTYVSQPAISYAAPLATK**TYVSQPALSY**AAPA**YAYHH

>|CPR6|

MAFKFVVFAALVAVARA (signal peptide)

GLIASPAVSYA**AAPA**YVA**AAPV**A**KVAYAAAPIAK**VAYAAQPEEYDANPQYSFSYGISDALTGDS**KSQQESRSGDVVQGSYSVVDPDGTKR**TVEYTADPHNGFNAVVH**R**EPLAA**KTIVAAAPVATKVIAQPAVAYAAPVAK**TISY**AAPV**A**KTISYAAPVATK**TYVAQPTLSY**AAPV**A**K**TYVSQPALSY**AAPA**YASYH

**END OF SEQUENCE CLUSTER**

>|CPR58|

MVARVCATLLLALAMFGSSALA (signal peptide)

**R**PGYAVDYYDHP**K**YAFNYGVADHSTGDV**K**SQHET**R**DGDVV**K**GQYSLVEPDGSV**R**TVDYTADPINGFNAVVS**K**TAPLVHAHAPVV**K**HVVPAV**K**AVHHVP**AAPV**VQ**K**VVYAHEPLLHVA**K**TGHYTEHAPAVHYGDYDGYYGHYDDSHYY

>|CPR59|

MAFFKSLVCLAVLGVAAA (signal peptide)

GVVPLATEYQGDYYAHP**K**YSYNYGVHDSLTGDV**K**SQVES**R**DGDVV**K**GQYSLVEPDGSV**R**TVDYTADDVNGFNAVVS**K**SAPSVHA**K**TVVAH**AAPA**VVAH**AAPA**VYASH**AAPA**VYASH**AAPA**VYASH**AAPA**VY**AAPA**VVA**K**TVVSH**AAPA**VYASH**AAPA**VYASH**AAPA**VYASHAYAPAHGTVEYHG**K**A**AAPL**AYYH

>|CPR70|

MKCFVAIALLAVAVSA (signal peptide)

SPVEYGHYAPHALVHAPVAVHAPVL**K**HVVAEPVAYP**K**YSFNYGI**K**DPHTGDI**K**SQAEE**R**DGDVV**K**GQYSLVEPDGSV**R**TVDYTADDHNGFNAVVH**K**SAPAVQ**K**VI**AAPV**AHYAPAPVLSHYVH

**3RA SEQUENCE CLUSTER**

>|CPR83|

MAFKLCVILAIMGVANA (signal peptide)

VLLPTLV**KK**VEVEAPAEYQFSYSVHDDHTGDI**K**SQQEE**R**HGDDV**K**GQYTLIDADGH**RR**VVDYTADEHNGFNAVV**RR**EPLEGH**K**LV**K**TVVPVH**K**VAVPVAHYVAPAL**K**VAVPVA**K**YAVPVA**K**VLAPAHVATVSFSAPSLTYHY

>|CPR84/CPR108| -- CPR84 Has an extra VPVAKVA (in bold and italics) relative to CPR108; unique peptide for that region was not found.

MAFKYCILFAVVAAASA (signal peptide)

AVLPVAV**K**HIEYADAPAEYQFEYSVHDDHTGDI**K**SQHEE**R**HGDNVVGQYTLIDADGY**RR**VVEYTADEHNGFNAVV**RR**EPV**K**TAIPVA**K**VVAPIA**K**VYAAPIA**K**VA***VPVAKVA***VPVA**K**VAYPAYHY

**END OF SEQUENCE CLUSTER**

>|CPR110|

MFKVFVVLAAVAVGVNS (signal peptide)

VAIGV**AAPA**TLV**K**TEEYDAHPQYSFSYDVQDSLTGDN**K**QQHET**R**DGDVVQGQYSLVEPDGT**RR**TVDYTADPVNGFNAVVS**K**SADAAVV**K**TVA**AAPV**AHVAYHAPTVAVAHAPAVAVAHAPVAYHAPAATAYVSHAPAYVSHAPAYVSHAPVYAHAPVVAHAPVLSHAVYH**R**

>|CPR114|

MISKVLLVASLVAASVA (signal peptide)

APVYGPLSYGPALSYGYAA**K**AIQPLHTVA**AAPV**LHAPAIAVA**AAPV**L**K**HVEAYDPNPHYSFSYGVSDPHTGDS**K**HAEETLSNGVVHGSYSLTEPDGTI**RK**VTYTAD**K**IHGFNAVVE**K**SGHAIHTAPVL**KK**VVA**AAPL**VHAALPYYHH

**2RB SEQUENCE CLUSTER**

>|CPR115|

MAFKFAVFAAIVAVASA (signal peptide)

VAIHPSPLGAYHAPLGAYHAPLGAYHAPLGAYHAPLGAYPAVA**KVAAPLAVAK**VAAPYADYDASPQYSYSYAVADAVTGDN**KNQQESRSGDVVTGSYSLVEPDGTRRTVEYNADPINGFNAVVHREPLAVKAVAPIAKYAAPLAYPAVAKVAAPYAPYGYPAYGK**AILG

>|CPR117/CPR154|

MAFKFAVFAAIVAVANA (signal peptide)

VAIGYPAPLGAYHAPLGAYPAVA**KVAAPVVAKVADDYDPNPQYSYSYHIADALTGDNKEQQESRSGDVVTGSYSLVEPDGTRRVVEYTADPVNGFNAVVHREPLAVKAVAPVAKIAAPLAYPAVAKVAAPYAPYGYPAYGK**AILG

>|CPR118/CPR119/CPR158|

MAFKFAVFAAIVAVASA (signal peptide)

VAIHPSPLGAYHAPLGAYHAPLGAYHAPLGAYHAPLGAYHAPLGAYPAVA**KVAAPLAVAK**VAAPYAEYDA**NPQYSYSYAVADAVTGDNKNQQESRSGDVVTGSYSLVEPDGTRRTVEYNADPINGFNAVVHREPLAVKAVAPIAKYAAPLAYPAVAKVAAPYAPYGYPAYGK**AILG

>|CPR120|

MAFKFAVFAAIVAVASA (signal peptide)

VAIHPSPLGAYHAPLGAYHAPLGAYHAPLGAYHAPLGAYPAVA**K**I**AAPL**AVA**KVAAPYAEYDANPQYSYSYAVADAVTGDNKNQQESR**SGDVVTGSYSLVEPDGT**RRTVEYNADPINGFNAVVHREPLAVKAVAPIAK**Y**AAPL**AFPAVA**KVAAPYAPYGYPAYGK**AILG

>|CPR121| -- differs from CPR118/CPR119/CPR158 ONLY in signal peptide

MVFKFAVFAAIVAVASA (signal peptide)

VAIHPSPLGAYHAPLGAYHAPLGAYHAPLGAYHAPLGAYHAPLGAYPAVA**KVAAPLAVAKVAAPYAEYDANPQYSYSYAVADAVTGDNKNQQESRSGDVVTGSYSLVEPDGTRRTVEYNADPINGFNAVVHREPLAVKAVAPIAKYAAPLAYPAVAKVAAPYAPYGYPAYGK**AILG

>|CPR122|

MALRFAVLAAFVATASA (signal peptide)

VAIGYPAPYGAYPAVA**K**V**AAPL**ADYDPNPQYSYSYAVSDALTGDN**K**SQQES**R**SGDVVSGSYSLIEPDGTQ**R**VVEYTADPVNGFNAVVH**R**GAGVV**K**AVAPVA**K**F**AAPL**AYPAVA**K**V**AAPL**YG

>|CPR123|

MAFKFAVFAAIVAVANA (signal peptide)

VAIGYPAPLGAYPAVA**KVAAPVVAKVADDYDPNPQYSYSYHIADALTGDNKEQQESRSGDVVTGSYSLVEPDGTRRVVEYTADPVNGFNAVVHREPLAVKAVAPVAKIAAPLAYPAVAK**VAAPYAPYGYPGYG**K**AILG

**END OF SEQUENCE CLUSTER**

>|CPR135|

MRAFVVATILCVGFVSA (signal peptide)

YPQQAVDPAYL**R**QYYQQIAQAAGAQNAAQG**R**ADATPIHEQGAQEQQHIPQYLPQGQPQ**R**TQYQQPQA**R**QYQPQYQPQQIQYVQEQQYQQPQPQL**K**VS**K**P**R**PQYLQGGQ**K**QPLEEEQEDYDANPSYQFGFDV**K**DDEFTNYQN**RK**EQ**R**DGNVI**K**GSYSVVDSDGFI**R**TVTYTADP**K**EGF**K**AEVS**R**QPTDIVV**K**IPTPAPQSQHD**R**FASQPQSAGAY**R**VQQQPQQQQQAQP**R**P**R**PQEYSQYQ

>|CPR140|

MKMFAAVLLAACLATSSLA (signal peptide)

SPAPDCPPSGHVSGYSYPAPTVQLSVG**K**AINSVSVTPGFSSYSVDGEV**K**YASVGPSYSSYEASPAYSAYGASGEDL**K**LTSYEAGV**K**TVE**AAPA**VTYTA**K**TAGVVYAD**K**SPAATFASVVPSVSYT**K**TV**AAPA**VYAQPAVS**K**VYTSE**AAPA**YQVT**K**EYLPPTV**K**TYATAPAVASYVSTPTVT**K**YA**AAPA**VSSYVATAPVVS**K**YVSAGPAVSYS**AAPV**A**K**VATYSSYAPAA**K**VATYATPAYGYAASYAPAV**K**ASYASYAPATSYASYAPATTYASYTPTA**K**VASYTPAVGYAAYAPAA**K**VAY**AAPA**ASYASSYATTS**K**LAY**AAPA**VT**K**TVVSAPAVASYYSAPAVT**K**YSSAPAVSTAYVSAPTVT**K**YAAAGVAYAPAVSSYVAPYSAQAYTPAVS**K**YVSTPAVSSYYATPAVS**K**VVSSPAYASYVSAPAVT**K**YASAPAVSSAYVSTPVVS**K**TAYSGAYL**AAPA**VA**K**VATAYGPAVASYSTGPAVSAYSTGSAYSSYSVAPAVS**K**VVSSPAVAAYSTGPAYSSYSVAPAVS**K**VYSTPAVAAYSAVPAVS**K**VYSTPAVASYSAVPAVS**K**VYSTPAVASYSASPAYSSYSATPAVASYSAVPAVS**K**VYSTPAVASYSAVPAVS**K**VYSTPAVASYSAVPAVS**K**YAA**AAPA**LTTAYTAPATVV**K**VYSSPAVAAYSAGPAYSSYSATPAVAAYSAGPAYSAYSVAPAVT**K**YATSGVAGYATSGATHGYYAAGPAVSAAHYGGY**R**YAA**AAPA**LTTAYTAPATVV**K**TVAPTTVV**K**TVAE**K**YLEHYEDNA**R**YAFEYGVNDPLTGDI**K**HQ**K**EE**R**DGDVV**R**GQYSLVEPDGNV**R**TVDYYADWATGFHATVTNS**R**DQVHAT**K**VLG**KR**DTV**K**A

>|CPR147|

MKIYVAVITIALIALAAT (signal peptide)

EPPAP**R**NGFTSSSSNYLPPNQSFNGNNGYNYNSNDNGYNYPSSSNGENYPATSQQYGPPLGNDGNGGYNYEDANVQPA**K**YSFEYNVQDFTSGNDFGHMES**R**DGD**R**TVG**R**YFVLLPDG**RK**QVVNYEADQNGY**R**PTITYEDIGTGNGANSNNGAYEGNGQFNGYQ

>|CPR160|

MASKNPSIAWIVCVVIASLTTKGYG (signal peptide) -- atypical R&R Consensus

WNLPASYYQGAGGAGAGGGYFGG**R**GESTLNNQGLAGASAGSWNAGGVGGGGGFPSQGQTGAGASSGVD**R**AASFGGNQWSAGGDG**R**GLGGGVAPGGVSTGGGDLGAGGTGFQQGGTGAGVGGSGSGAPLGAGSGSGFGYNGQAGGFGGSNFGQGQYAPHNTNGVASGASGFGGSFGGSGGNYGPSGHHHGY**R**HPTTYAG**R**PMTLVYPAGGWPWHGVGYVY**R**VPVAHINPDGSYGFSYYTPNSA**R**DETGHANGNVEGTYGFQNDGA**K**HNFSFNAAPDVDL**R**SSIGDT**R**LGGLNPDEYGPQSVHS**R**G**R**LPLIPATSFDGAADEQT**R**TADGLPVSVEQSTGANSWST**R**SGIDGAGSEGGSDDSSLSVVGLP**K**ATTEAGDASQPTA**R**DQVV**R**GSTEHSANELDG**RR**LGGFDGTATTV**R**PLGGSVEQVGA**R**PVNDVPSYSNEID**R**NGVQYNGGVGIVPVD**R**SYQFGYQTPDAT**R**EESADQAGNV**R**GSFSYNNEAG**R**NDLQYVAGTGMGF**R**PTGGSLSVPNGLPGDNGQ**R**FGPATGAGGVFGVDGG**R**SLGAGSQGAQVGGGLGGAFGGSDAGFVGDG**R**SVATGDQGAPLGGTAGSGFGMDG**R**SLAFGSQGSQLGGGVGGGFGTGVGNGFGADG**R**PLGSGTGVGVDG**R**SFGSQPGGGFGAGSGAGFGADG**R**PLGFGNQGTQTVSGTGSGAGFGADG**R**PLGFGNQGTQSGGDGVGGAFGT**R**SGSGFGAGGFGNQGASGAAFGADG**RR**LGFGNQGTQFGAGGNGASFGGSDG**R**SFGFGNQGGQGFATAD**R**GLQAATT**R**NPVT**R**SPSAGSFDGSGLSSADEGEESTTLNAPDDSEQ**R**NTFGGFGDVS**R**AGLFTNAN**RR**LVQ

>|CPR162|

MWLTLAFGLVVLVASGDA (signal peptide) -- atypical R&R Consensus

QT**RR**L**R**V**R**P**R**VLAAPSSAEYVDSAEDAQQDN**R**QYYAAPQ**R**AQE**R**LGDVVLVASSDEDYGGGQYGAPVAAA**R**P**R**ADQQQYQ**R**PAA**K**STT**AAPV**AA**R**Q**K**APASES**R**APPVQTI**R**NYS**K**VNDDGSFTFGYEAADGSF**K**EET**R**GTDCVV**R**G**K**YGYIDPDGN**KR**EFTYVSGNPCDPNNPDGSEEEESD**R**AEGGQEDSNENVPQNYPV**RR**PVPVA**R**PTP**AAPVR**HHSTPAPAP**R**PTTTVFQNDYQD**R**Q**R**QEQQSADAEEEVQIGQ**R**GSPP**R**PAA**R**PFAGAVTTTQ**R**P**R**VQIVSTTPSPTPTIFHSP**AAPA**APQTVLPVNITP**K**PVY**R**VSPLPTQPTLAPTTY**R**PTSSPST**R**GPTGSIDFEAEF**KR**FQADN**K**LPSPPTPSTAPSGGAAP**K**PTGSPFG**R**PGPQLAAGNPIYQSQLIFDPASGQYDTALYQQLPQSDGDFQLNH**R**IQPYVAGPQQHQHHQPQPQPQQQQHPGAGQLVTLEQLQQQSPLY**R**AQPSP**R**PATVQIPQQLYQ**K**QQNELQFINSQQLFAQQLELQQSQL**R**AD**R**LEAA**KK**VTVGGPPMH**R**FQPQPQPQQQYYFIQPQGPPQGAPGQIDAFL**R**GHNIEY

>|CPR163|

MKYTLALLPLAGLITLATA (signal peptide)

QYGPAPP**R**LNIPGAIPLPPI**R**EE**R**LLPQQSPQVI**R**V**RR**PGAV**R**IAAPNAIHHQLPSALP**K**FHDIPSTVEH**K**PVTEEPEDDF**R**PAFIPQLQQPHHHHPSPATLASPAAPSPAASPALQFPIPADEQPSE**R**D**R**ELQQNVLS**R**FNAQEN**R**PAPIQ**R**AQIPE**R**FFATD**K**EPA**R**FPAE**R**PQPQQQQQQPTP**K**QY**R**PAPQQIA**R**PAPIAAPQQSF**R**QQQHFQDED**RR**PAPHQQAA**R**PHTQQDQD**R**Q**RK**PVAQIL**RK**W**R**EEHEDGSITWGFENDDGSF**K**EETIGIDCVT**R**G**R**YGYVDPDGE**KR**EYTYETGIQCDPNQ**R**DEDDEDNLEVDYQEN**K**AVLPNGV**R**LDLNNMG**KK**QS**KR**PGGQQQQQQQQQYY**R**N

**NON-CPRs FOUND IN BOTH SOLUBLE FRACTION AND FINAL PELLET**

>|CPAP3-C-PA| -- differences between isoforms are italicized and bolded

MKYSIVFVVALFGAAVA (signal peptide)

QESF**K**CPDDFGFYPHHSSCD**K**YW**K**CDNNVAEL**K**TCGNGLAFDATDS**K**YLTENCDYLHNVDCGD**R**TQLEPPISTPHCE**R**LYGIFADAA**K**CDVFWNCWNGEAS**R**YQCSPGLAYD**R**EA**R**VCMWADQVPEC**K**NEEVANGFACPAAGEISNAGSFS**R**HAHPEDC**RK**YYICLEGVA**R**EYGCPIGTVF**K**IGDADGTGNCEDPEDVPGCEDYYGD***QDI****KALQKKGY*

>|CPAP3-C-PC| -- differences between isoforms are italicized and bolded

MKYSIVFVVALFGAAVA (signal peptide)

QESF**K**CPDDFGFYPHHSSCD**K**YW**K**CDNNVAEL**K**TCGNGLAFDATDS**K**YLTENCDYLHNVDCGD**R**TQLEPPISTPHCE**R**LYGIFADAA**K**CDVFWNCWNGEAS**R**YQCSPGLAYD**R**EA**R**VCMWADQVPEC**K**NEEVANGFACPAAGEISNAGSFS**R**HAHPEDC**RK**YYICLEGVA**R**EYGCPIGTVF**K**IGDADGTGNCEDPEDVPGCEDYYGD***LDLKSIRKSELLAGLALQSGGAPAATKANVKSNRPAPKDSN***

>|CPLCG4|

MKVAVVAVVLALAVVSEA (signal peptide)

GVLPWGWPYAGLPAAYPVAAWPPAAIHAAYPAYAHHGAYLAAPHAAILAAPHAPAASVAHHAGVVPGATSVTAT**R**GAVHVAPLPGHAVSQQQLNLAPAPGTI

>|CPLCG5|

MKCIVAAVAVIALAVAAEA (signal peptide)

GYPYAGYPYAGYPYAGYGATVVQANAGAWPYAHAAYPYAHAGYPYAHAAAYPAAVAAYAAPHAALLAAPHAPLASVAHHAGVVPGATSVTAT**R**GAVHVAPLPGHAVSQ**K**QLNLAPAPGTL

>|CPLCG15|

MKCIAAVVMMAVAVAVQG (signal peptide)

TSVTYWGNGAALQHLAPVSVGSWVQDNSYA**R**IVSPWNYAYQAPVATVA**AAPV**AYAAPYTYQPAVAVVAQ**K**EA**R**YLAAN**R**GAVHEAPLPGHVVNQQSLNLEPAPGTL

>|CPLCX3|

MFKLVVLPLFFAAVSA (signal peptide)

GYLGSPLAYSAPAYAHAPLAAAYHAPYAYGAPVV**K**TV**AAPV**AY**AAPA**YHAAPIV**K**AVAPVATSYANTY**K**VSV**K**APVAY**AAPA**VVSHAPVAY**AAPA**YAAHAY**AAPA**YAAHAY**AAPA**YAHGYYH

**NON-CPRS FOUND ALMOST EXCLUSIVELY ONLY IN FINAL PELLET**

>|CPAP1-G|

MEEEALVALTLQLCCGILLLCVVSG (signal peptide)

QQY**K**QQAGSPASSASSAASSSSDAVPAAANNVAY**R**A**R**PYSNQYSSSSSEEEEDD**R**PVASYNSG**R**QQQQGNTL**KK**SF**KK**PSYSSEELEQEEEPD**R**LTLLLE**K**SQFQCTG**R**TTGYYADESLGCEVFHYCQENQ**K**HSWICPEGFTFHQVHLICMPPSGDNICEQSS**K**YHFVNDYLY**K**PINMEEHMT**K**PNVTL**R**YSE**R**YYPENFYVDE**R**HYDEE**R**VL**R**QHEE**R**HQPQQPI**K**QTYHHQPQQQTI**RK**QPVYATTPSSY**R**LPSSPQPTHSVY**R**SPDEINISLQQ**RR**PAAQPGSYIQSTTP**R**YEDESEYDSYE**RK**

>|CPF2|

MAFKFVVFLASLAVASA (signal peptide)

GYLEADHAVQY**AAPV**AHYSPASAVSYSTISQ**AAPAK**LAY**AAPV**A**K**TVSYAAPQVYAAPQVYA**AAPV**T**K**TYVSSPAVGATHESTI**R**SHDSTISHYS**K**AVDTPYSSV**RK**SDT**R**ITNELP**K**YAYAQPVLA**K**QVAYA**AAPA**VHTTY**AAPA**AVHTSYAH**AAPA**VTYAH**AAPA**YQTYAH**AAPA**AVHTSYTH**AAPA**VHATY**AAPA**VQTYAH**AAPA**VHTTY**AAPA**VHTSYAH**AAPA**VHATY**AAPA**AVQTYAH**AAPA**VHTST**K**TLTYSPAVQVAHTTYEDAHAHYAW

>|CPF3|

MY**R**FVALFALVAVSQA (signal peptide)

AYTLNPAGPTYAGIHTPAITSQQSNIL**R**SYGNLGQISTYS**K**TIDTPYSSVS**K**SDV**R**VSNPGLAVGHIAASYPHPI**AAPA**YGHVGYAATAL**K**NPALLGVAYS**AAPA**VAHMTYSNGLGINYAW

>|CPF4|

MAFKFVILAALVAAVSA (signal peptide)

GGPAAYSIAAPSADFHSVGASHEHTV**K**GLYGQNVLSQYS**K**AVDSAHSSV**R**VHSS**R**LSNDGYAY**AAPA**V**K**Y**AAPA**YAAHY**AAPA**VHYPAAAHY**AAPA**VHYPAAAHY**AAPA**VHYAAHAPIV**K**AAYPAAY**AAPL**AY**K**TPL**AAPV**AAVHGGSVVQFAGLGASYAW

>|CPFL1|

MAFKIVVLFATLACASA (signal peptide)

GYVEPEHHHLSYA**AAPV**AHYSSAPAVSYSSIT**R**HETP**K**VAVA**K**QVTYAEPAVHY**AAPL**T**K**TYAVHEPAL**K**TVVAQPAYT**K**TVYAQEPAHVYAH**AAPV**VAA**K**TVSYAAPQVHYQAAPQVHYQ**AAPA**LV**K**NVEYT**K**TLAYAPVT**K**TLVSEPTYT**K**HVVAEPTYT**K**TLLAQPAYT**K**YVSQPTYT**K**TLVAEHQPLYHHQPAVYAH**AAPV**VAA**K**TVSY**AAPA**AHVSHVSYADNAAHYAW

>|CPLCA1|

MFRLVVLSVVLAVAAA (signal peptide)

APGAHLVHSAPLAYSTVVA**AAPA**LVAQ**K**EISYQ**K**SIVEEPTVAHVGTIE**K**SVPTGYSHQSFTQYHN**K**QVAEPVFAPAV**KK**TVVSTPVE**K**TTYVQA**AAPV**VH**AAPV**VY**AAPV**QTVY**AAPV**A**K**TYAY**AAPV**E**K**TYTY**AAPV**AISYE**AAPV**AY**AAPVK**TSYVSSYPSVY**AAPV**VYAHDY

Most peptides were shared with CPLCA2 that had no unique peptides.

>|CPLCA3|

MLKLVVLSAVLAVVAA (signal peptide)

**R**PGALTYSAPLAYAPATLIA**K**PEIYYQ**K**SIIEEPTVAHVGSLV**K**TIPTAVSHQSSTVVHNSA**K**ITEPIYAPAV**K**QTLVSTPIA**K**TTYF**AAPA**AYAY**AAPA**LAYHDAYAYHHL

>|CPLCG14|

MKCIIVAVIVALAVAAEG (signal peptide)

YGVSSYAVPLAYSVPQTTVVQQNVAP**K**YVVSGYAAPYVASPYVAAAHSYAVPAAVSYASTAVHAVPAVTYAAPHATVY**AAPV**QQEA**R**YVAAN**R**GAVHEAPLAGHAVNQQSLNLAPAPGTL

>|CPLCP8|

MKGFVVFVMAIALVAS (signal peptide)

AEI**KKK**DAEAPAEAEANGE**KK**QE**KR**GLWDLGYGYESHGWDSH**K**SHGWEEPHVTTIT**KK**VHVPYPVEVE**K**HVPYPV**K**VPYPVTVE**K**HVPVVVE**KK**VPVYVE**K**HVPVHVD**R**PVPYPV**K**VPV**K**VVH**K**EYVEVP**K**PYPVHVE**K**HVPVVV**KK**PVYVE**K**HVPVVV**K**SHGWEPHSHSYSEFHSW

>|CPLCP12|

MKFFICLSALLLVSANA (signal peptide)

ESEQ**KK**SAADS**K**AAVPLE**KK**LD**KR**GLLSLGYGYGINGLDVGYIGGGHLGGAYHEAHDHHYGHGVYLGGHTDVT**K**TVTLV**K**GVPVPYTVE**K**HVPYTVE**K**HVPYPV**K**VPVPQPYEVV**K**HVPVHV**K**EYV**K**VPVHVPQPYPVE**KK**VPYPVHVPVD**R**PYPV**K**VFVPQPYEVT**K**HVPYPV**K**VPVPQPYEVT**K**HVPVPV**K**VEVPVPVPYTVE**KK**VPYPV**K**VPVD**R**PYPVHVPAPYPVEVE**K**PVPYTVE**K**PVPYEV**K**VPVD**R**PYPVPVE**K**PVPYPV**K**VPVP**K**PYYVE**K**HIPYTVE**K**PVPVPV**K**VPID**R**PYPVTVE**K**HIPVE**K**PVPVPV**K**VPVAVPVPVHHDHHHHHLEHLHEPHHHHHHDVSYTSFSGYGQDYSYHH

>|CPLCX2|

MYKLFVVACFLAVAAA (signal peptide)

APGVVLPAHTVVHSAPLVH**AAPV**AVSHSSSHVVHHAPVV**K**TVAVHSAPVVAVHHAPVV**K**ALPVVHHAPVVHHAPVL**K**TVVHSAPVVHSVHPVPVV**K**SVHVASPVLVHH

>|CPLCX4|

MKILLLLGLIAAVRA (signal peptide)

APGYYGDHGLSYVA**AAPA**PYV**K**Y**AAPA**VSVVH**AAPA**PVL**K**YAVAPAPIV**K**AVAPAATSYATIHQVHAPVVH**AAPV**V**K**Y**AAPA**PVVSYVHSAPAVVAHAPAPLL**K**YAPAYHGW

>|CPLCX5|

MFTKLICIAALAISCASA (signal peptide)

**K**PGLLAPV**AAPV**AYAAGPAVVTAQSSQVVA**R**NYNGIAPLAYTAPAVAY**AAPA**VA**K**V**AAPL**AY**AAPA**PLAAAAYA**AAPL**AAPYVAPYAAPYAAPYAAAYAAPYAAAYAAPYA**K**AFASPLLA**R**APYQLW

>|CPLCX10| (AGAP006970)

MLKKTIVLSCLVAVVLC (signal)

AADLETAETSWGGGGGGGWSSGGGGGGWSSGGGGGYGGG**KK**VIIISSGGGGHGGSGGWSSGG**R**SLGGGGGWSSGGSGLG**K**GWSSGGGSYGGGGSYGGGGYS**K**GWPVS**K**GWSSGGSSFGGGYGGGSSFGGGHSGGGWSSGGSSLGGWPSGGGVS**K**GWPSSGGGYGGSQGWSSGGGYGGGSHGGWSSGGSGLGGGYGGGGGGWSSG**R**SLGGSGGWSSGGLSS**K**GWPSSGGS**K**GWPSGGYGGGSSGWSSGGSGWSGSSGW

>|TWDL1|

MKTFVLVSCCLVLASA (signal peptide)

**R**PEAGYSYN**R**PSTGGSFGGGSQQAIIQ**K**HIYVHVPPPEPEEV**R**VQ**R**PIQLAAPQ**K**HY**K**IIFI**K**APSAPSYQAPQIPIQPQNEE**K**TLVYVLV**KK**PDEQQDIVIPTPAPTQPS**K**PEVYFI**K**Y**K**TQ**K**ESSGGAASGGYASAGGFGGDLGGGLGGGDLGGHGSLVGGDLGGHGGHGGHGGDLGGHGGSGASAPAAQYGPPG**K**SGPY

>|TWDL9|

MKVLVVLACVAIVVA (signal peptide)

**R**PEPPVGGYSHHHHGGHGGHSGYNYNAPVPAHTQPFGHQAQFSGVAQSAGGSFSDTLSGAVTGSFSGIANTLSAGNTFTNANSFSNANAFTQSNANAFTQSNANAFSNAGSFVAPQQQIVQ**K**HIYVHVPPPEPQQSFQQQIVAPGL**R**Q**K**HY**K**IIFI**K**TPHQQPSAAQLALQQSQTEE**K**TIVYVLV**KK**PEAVGDISLPLPAVT**K**PS**K**PEVYFI**K**Y**R**TNTEEVQAPVGLNVGSSATAQADAGGLGFLSSSGAAQSSAVAQSAAVAQSSAVAGAHSHSNVPAQQYGTPAHHHGGYH

>|TWDL11|

MKVFVVLSVVLACAAA (signal peptide)

**R**PEAGFSSYSSAPSFTSVGDFGGPYSGSSSGGSSGYNYNPAPQIVQ**K**HIYVHVPPPE**K**EEVHYP**R**VSPVAPAQ**K**HY**K**IIFI**K**APSPPAP**K**APIIPVQPQNEE**K**TLVYVLH**KK**PEEPQDIVIPTPPPT**K**PS**K**PEVYFI**K**Y**K**TQ**K**EQSQP**K**PEYGPPGQSGPY

>|TWDL12|

MKSLLILFACIVCALA (signal peptide)

**R**PEPEPP**R**A**R**IVVPA**K**QQQLPQYEYGAP**K**PEYGPPAEEYGPPPPTVYGPPA**R**EYGPPP**K**LIT**K**NVYVHVPPEEPTEIV**K**SPVLEAPIP**KK**HY**K**IIFI**K**APAPPAPI**K**QVIPPQPQDEH**K**TLVYVLV**KK**PEDPAPLEIPVPETTEPN**K**PEVYFI**K**Y**K**EGE**K**EPH**K**QYGPPAPAYGPPSGPA**R**YQQF
