# Supplementary material for: Properties of the cuticular proteins of Anopheles gambiae as revealed by serial extraction of adults
Source: PLoS One. 2017 Apr 18;12(4):e0175423. doi: 10.1371/journal.pone.0175423 (PMC5395146; doi:10.1371/journal.pone.0175423)
Supplement: S1 Table — (DOCX) [file pone.0175423.s006.docx]

**S1 Table Description of samples from each extract used for LC-MS/MS analysis**

**Nomenclature reflects that used for data submitted to MassIVE.**

|  | PBS | EDTA | UREA -- 2M, 4M, 8M | SDS | FINAL PELLET |
| --- | --- | --- | --- | --- | --- |
| Batch 1 | entire gel  (S1, S2) | entire gel  (S1, S2) | entire gel (S1, S2)  gel slices A-D  ACN entire gel  (not 8M) | entire gel (S)  gel slices A-B | FP |
| Batch 2 | entire gel (S)  ACN entire gel | entire gel (S)  ACN entire gel | entire gel (S)  gel slices A-D  ACN entire gel | entire gel (S)  gel slices A-D  ACN entire gel | FP |
| Batch 3 | entire gel (S)  gel slices A-D | entire gel (S)  gel slices A-D | entire gel (S)  gel slices A-D | entire gel (S)  gel slices A-D | FP |

“Entire gel" - all of the short run gel area (three lanes) containing the proteins was used as a single sample for

LC-MS/MS analysis. When there were two replicates they were designated S1 and S2.

"Gel slices" - the gel area containing the proteins was horizontally divided into two (A, B) or four (A, B, C, D) equal parts, each part was used individually for LC-MS/MS analysis. Section A was at the top of the gel.

"ACN" - Acetonitrile treated samples, entire short run area with proteins.

See Experimental Methods (Preparation of samples for LC-MS/MS) for more details.
