# Supplementary material for: Properties of the cuticular proteins of Anopheles gambiae as revealed by serial extraction of adults
Source: PLoS One. 2017 Apr 18;12(4):e0175423. doi: 10.1371/journal.pone.0175423 (PMC5395146; doi:10.1371/journal.pone.0175423)
Supplement: S3 Table — Solubility classes and properties of CPs. (DOCX) [file pone.0175423.s008.docx]

**S3 Table Continuation of Text Fig 1. Solubility classes and properties of CPs.**

Data in the first 4 columns summarize data from this analysis. Parentheses around a + indicate that assignment to solubility group had minor exceptions. Proteins identified with three or more peptides are indicated with ++. Data entry required that at least one peptide for a protein was found in two of the three biological replicates. Details are in S3_Table.

Names of CPR proteins in the RR-1 group have a green background, RR-2 have a brown background. Names underlined scored below recommended threshold in CutProtFam-Pred. Two CPR sequences in small type and with no background color, did not score as either group but had a region recognized as pfam00379. Protein names separated by a / had identical sequences.

| Sequence Cluster/  Protein name | Soluble only | Both | Final pellet only | RT-qPCR data^a^ | | | RNAseq  rank  order^b^  5 da ♀♀ | Adult  structures where found^c^ | Cuticle location^d^ |
| --- | --- | --- | --- | --- | --- | --- | --- | --- | --- |
|  |  |  |  | **P12** | **P24** | **A**  **0-12** |  |  |  |
| CPR8 | **++** |  |  | **++** | **+++** | **-** | 45 | none |  |
| CPR15 | **(+)** |  |  | **-** | **+++** | **+** | 12 | JAELW |  |
| CPR23 | **+** |  |  | **-** | **+++** | **+** | **-** | AE |  |
| CPR26 | **++** |  |  | **-** | **+** | **+++** | 18 | JEL |  |
| CPR30 | **++** |  |  | **-** | **+++** | **-** | 28 | E |  |
| CPR75 | **+** |  |  | **-** | **-** | **+++** | 4 | E | soft |
| CPR76 | **++** |  |  | **-** | **-** | **+++** | 8 | none |  |
| CPR78 | **+** |  |  | **+++** | **++** | **++** | - | none |  |
| CPR81 | **++** |  |  | **+** | **+++** | **+++** | 11 | none |  |
| CPR106 | **+** |  |  | **-** | **+++** | **-** | **-** | none |  |
| CPR69 | **++** |  |  | **-** | **+++** | **-** | **-** | L |  |
| CPR128 | **+** |  |  | **+** | **+++** | **-** | **-** | none |  |
| CPR132 | **++** |  |  | **-** | **++** | **+++** | 3 | EL |  |
| CPR16 |  | **++** |  | **-** | **+++** | **+** | 6 | JAELW |  |
| CPR62 |  | **++** |  | **+** | **+++** | **-** | **-** | JEL |  |
| CPR10 |  | **++** |  | **-** | **+++** | **+++** | 2 | JAEL |  |
| CPR116 |  | **+** |  | **-** | **+++** | **-** | **-** | JE |  |
| CPR124 |  | **++** |  | **-** | **+++** | **-** | **-** | JE |  |
| CPR130 |  | **++** |  | **+** | **+** | **+++** | **-** | JAELW |  |
| CPAP3-C-PA |  | **++** |  |  |  |  | **-** | JAELW |  |
| CPAP3-C-PC |  |  |  |  |  |  |  |  |  |
| CPLCG4 |  | **+** |  | **-** | **+** | **+++** | **-** | JAELW | endo |
| CPLCG5 |  | **++** |  | **-** | **+** | **+++** | 37 | JAELW | endo |
| CPLCG15 |  | **+** |  | **-** | **-** | **-** | **-** | AELW |  |
| CPLCX3 |  | **+** |  | **-** | **+++** | **-** | **-** | JAELW |  |
| CPR9 |  |  | **++** | **+++** | **++** | **++** | 14 | JEW |  |
| CPR12/13 |  |  | **+** | **-** | **+++** | **-** | **-** | JE | soft |
| CPR113 |  |  | **++** | **+** | **+++** | **+** | 10 | JAE |  |
| CPR125 |  |  | **++** | **+++** | **+** | **+** | 40 | JAELW | hard, endo |
| CPR126 |  |  | **++** | **++** | **+++** | **+++** | **-** | JAELW |  |
| CPR127 |  |  | **++** | **+++** | **+** | **++** | 23 | JAELW |  |
| CPR151 |  |  | **++** | **+++** | **+** | **-** | **-** | JEL | soft |

| 2RA | CPR1 |  |  | ++ | P0 only | | | - | JAEW | exo + endo |
| --- | --- | --- | --- | --- | --- | --- | --- | --- | --- | --- |
|  | **CPR2/4** |  |  | **++** |  |  |  | **-** | JAEW |  |
|  | **CPR3** |  |  |  |  |  |  | **-** | JAEW |  |
|  | **CPR5** |  |  |  |  |  |  | **-** | JAEW |  |
|  | **CPR6** |  |  |  |  |  |  | **-** | JAEW |  |
| CPR58 | |  |  | **++** | **+++** | **++** | **-** | **-** | JAELW |  |
| CPR59 | |  |  | **(++)** | + | **+++** | **+** | 36 | JAELW | exo + endo |
| CPR70 | |  |  | **++** | +++ | ++ | **-** | 22 | JAELW |  |
| 3RA | **CPR83** |  |  | **++** | **-** | **+++** | - | - | EL |  |
|  | **CPR84/**  **CPR108** |  | | **++** | **-** | **+++** | - | - | E |  |
|  |  |  |  |  |  |  |  | **-** |  |  |
| CPR110 | |  |  | **++** | **+** | **+++** | **-** | **-** | JAELW |  |
| CPR114 | |  |  | **++** | **+++** | **-** | **-** | **-** | JAELW |  |
| 2RB | **CPR115**  **CPR117/154** |  |  | (++) | **+** | **+++** | - | **-** | JAELW | exo |
|  |  |  |  |  | **-** | **+++** | **-** | **-** |  |  |
|  | **CPR118/119/**  **121/158** |  |  | **+** | **+** | **+++** | **-** | **-** |  |  |
|  |  |  |  |  | **+++** | **++** | **-** | **-** |  |  |
|  |  |  |  |  | **+** | **+++** | **-** | **-** |  |  |
|  | **CPR120** |  |  | **+** | **+** | **+++** | **-** | **-** |  |  |
|  | **CPR122** |  |  | (++) | **-** | **+++** | **-** | **-** |  |  |
|  | **CPR123** |  |  | **+** | **-** | **+++** | **-** | **-** |  |  |
| CPR135 | |  |  | **++** | **+++** | **-** | **-** | **-** | JAELW |  |
| CPR140 | |  |  | (**++**) | **++** | **+++** | **-** | **-** | JAELW | mainly exo |
| CPR147 | |  |  | **+** | **++** | **+++** | **-** | - | J |  |
| CPR160 | |  |  | **++** |  |  |  | 9 | JAELW |  |
| CPR162 | |  |  | **++** |  |  |  | **-** | JAELW |  |
| CPR163 | |  |  | **++** |  |  |  | **-** | JAELW |  |
| CPAP1-G | |  |  | ++ |  |  |  | **-** | JAE |  |
| CPF2 | |  |  | **+** | **+++** | **-** | **-** | **-** | AW |  |
| CPF3 | |  |  | (**++**) | **-** | **+++** | **-** | 27 | JAELW | exo |
| CPF4 | |  |  | **++** | **-** | **+++** | **-** | **-** | JAELW |  |
| CPFL1 | |  |  | **++** | **-** | **+++** | **-** | **-** | JAE |  |
| CPLCA1 | |  |  | **++** | **++** | **-** | **+++** | **-** | JAEL |  |
| CPLCA3 | |  |  | (**+**) |  |  |  | **-** | JAELW |  |
| CPLCG14 | |  |  | **+** | **+** | **+** | **+++** | **-** | AW |  |
| CPLCP8 | |  |  | **++** | **+++** | **+** | **-** | **-** | JAL |  |
| CPLCP12 | |  |  | **++** | **+++** | **+** | **+** | **-** | JAELW |  |
| CPLCX2 | |  |  | **++** | **+** | **+++** | **-** | 7 | JAEW |  |
| CPLCX4 | |  |  | **++** |  |  |  | **-** | EW |  |
| CPLCX5 | |  |  | **+** |  |  |  | **-** | JE |  |
| CPLCX10 | |  |  | **++** |  |  |  | 19 | JAEL |  |
| TWDL1 | |  |  | **++** | **+++** | **-** | **-** | **-** | JAELW |  |
| TWDL9 | |  |  | **+** | **+++** | **-** | **-** | **-** | JAEW |  |
| TWDL11 | |  |  | **++** | **+++** | **+** | **-** | **-** | JELW |  |
| TWDL12 | |  |  | **++** | **-** | **+++** | **-** | 25 | JL |  |

No expression or location data were available for blank cells.

^a^Togawa et al., 2007; Togawa et al., 2008; Cornman and Willis 2009 RT-qPCR data scored as; (+++) maximum; (++) at least 50% of maximum; + (10-50%) of maximum; (-) less than 10% of maximum. Number of + is not related to actual values that differed by >10,000 for different genes; rather that is shown by the color of the symbol. Plusses in red have a value >10,000, in blue 1,000-9,999, others were less than 1,000.

^b^Vannini et al., 2014a. Rank orders for all CP genes with FPKM >1.0 in non-blood fed females 5-6 days after eclosion.

^c^Zhou et al. 2016 Data are LC-MS/MS. Abbreviations for adult structures are: J - 2^nd^ antennal segment with Johnston’s organ; A - rest of antenna; E - corneal lens of compound eye; L - leg; W - wing.

^d^Vannini et al., 2014b, 2015; Vannini and Willis, 2016 a, b. Data are EM immunolocalization.
