# Supplementary material for: Properties of the cuticular proteins of Anopheles gambiae as revealed by serial extraction of adults
Source: PLoS One. 2017 Apr 18;12(4):e0175423. doi: 10.1371/journal.pone.0175423 (PMC5395146; doi:10.1371/journal.pone.0175423)
Supplement: S8 Table — (DOCX) [file pone.0175423.s013.docx]

**S8 Table Statistical Analyses of Data in Fig 1 and S3 Table**

F tests carried out on SAS PROC GLM.

| Source | pI | Histidine (H) | | Tyrosine (Y) | | Glutamine (Q) | | | length | | RT-qPCR | |
| --- | --- | --- | --- | --- | --- | --- | --- | --- | --- | --- | --- | --- |
|  | *p* | *p* | | *p* | | *p* | | | *p* | | *p* | |
|  |  | % | # | % | # | | % | # | | # aa | | Peak time |
| SOLUBILITY | **.0007** | .1135 | .1805 | .0812 | .0979 | | .9217 | .4993 | | .4175 | | .**0005** |
| CP Family (RR-1, RR-2, other) | **<.0001** | .1571 | .4301 | **.0085** | .9636 | | **.0170** | **.0054** | | **.0002** | | .3764 |
| CP Family*solubility | .5884 | .9675 | .2512 | .**0219** | .8602 | | **.0067** | **.0133** | | .7482 | | .7868 |
| FP RR-1 vs RR-2 | .9902 | .3433 | .9536 | .6286 | .5101 | | .1872 | .2838 | | .2587 | | .8028 |
| FP CPRs vs non-CPRs | .3315 | .6794 | .8841 | **.0380** | .1230 | | .7422 | .6936 | | .9520 | | .4943 |

t tests carried out using SAS.

| Comparison | pI | Histidine (H) | | Tyrosine (Y) | | Glutamine (Q | | length | RT-qPCR |
| --- | --- | --- | --- | --- | --- | --- | --- | --- | --- |
|  | *p* | *p* | | *p* | | *p* | | *p* | *p* |
|  |  | % | # | % | # | % | # | # aa | Peak time |
| Sol 1 V sol 2 | **≤ .01** | **≤ .01** | **≤ .01** | > .05 | > .05 | > .05 | > .05 | > .05 | > .05 |
| Sol 1 V sol 3 | **≤ .01** | **≤ .01** | **≤ .01** | > .05 | **.05 <p <.01** | > .05 | > .05 | > .05 | **≤ .01** |
| Sol 2 v sol 3 | **.05 <p <.01** | > .05 | > .05 | > .05 | > .05 | > .05 | > .05 | > .05 | **≤ .01** |

Sol 1 = soluble; Sol 2 = both soluble and final pellet; Sol 3 = final pellet only

SAS Proc Freq Procedure

| CP Family |  | Soluble Only | Both soluble and FP | Final Pellet Only |
| --- | --- | --- | --- | --- |
| RR-1 | observed | **10** | 2 | **7** |
|  | expected | **3.34** | 2.82 | **12.84** |
| RR-2 | observed | 3 | 4 | 25 |
|  | expected | 5.62 | 4.76 | 21.62 |
| Other | observed | 0 | 5 | 18 |
|  | expected | 4.04 | 3.42 | 15.54 |
